# Loss of RNA expression and allele-specific expression associated with congenital heart disease

**DOI:** 10.1038/ncomms12824

**Published:** 2016-09-27

**Authors:** David M. McKean, Jason Homsy, Hiroko Wakimoto, Neil Patel, Joshua Gorham, Steven R. DePalma, James S. Ware, Samir Zaidi, Wenji Ma, Nihir Patel, Richard P. Lifton, Wendy K. Chung, Richard Kim, Yufeng Shen, Martina Brueckner, Elizabeth Goldmuntz, Andrew J. Sharp, Christine E. Seidman, Bruce D. Gelb, J. G. Seidman

**Affiliations:** 1 Department of Genetics, Harvard Medical School, Boston, Massachusetts 02115, USA; 2 Cardiovascular Division, Brigham and Women's Hospital, Harvard University, Boston, Massachusetts 02115, USA; 3 Cardiovascular Research Center, Massachusetts General Hospital, Boston, Massachusetts 02114, USA; 4 The Mindich Child Health and Development Institute, Icahn School of Medicine at Mount Sinai, New York, New York 10029, USA; 5 Howard Hughes Medical Institute, Harvard University, Boston, Massachusetts 02115, USA; 6 National Institute for Health Research Cardiovascular Biomedical Research Unit at Royal Brompton and Harefield National Health Service Foundation Trust and Imperial College London, London SW3 6NP, UK; 7 National Heart and Lung Institute, Imperial College London, London SW3 6NP, UK; 8 Department of Genetics, Yale University School of Medicine, New Haven, Connecticut 06510, USA; 9 Department of Systems Biology, Columbia University Medical Center, New York, New York 10032, USA; 10 Howard Hughes Medical Institute, Yale University, Connecticut 06510, USA; 11 Department of Pediatrics and Medicine, Columbia University Medical Center, New York, New York 10032, USA; 12 Section of Cardiothoracic Surgery, University of Southern California Keck School of Medicine, Los Angeles, California 90089, USA; 13 Department of Biomedical Informatics, Columbia University Medical Center, New York, New York 10032, USA; 14 Department of Pediatrics, The Perelman School of Medicine, University of Pennsylvania, Philadelphia, Pennsylvania 19104, USA; 15 Department of Genetics and Genomic Sciences, Icahn School of Medicine at Mount Sinai, New York, New York 10029, USA; 16 Department of Pediatrics, Icahn School of Medicine at Mount Sinai, New York, New York 10029, USA

## Abstract

Congenital heart disease (CHD), a prevalent birth defect occurring in 1% of newborns, likely results from aberrant expression of cardiac developmental genes. Mutations in a variety of cardiac transcription factors, developmental signalling molecules and molecules that modify chromatin cause at least 20% of disease, but most CHD remains unexplained. We employ RNAseq analyses to assess allele-specific expression (ASE) and biallelic loss-of-expression (LOE) in 172 tissue samples from 144 surgically repaired CHD subjects. Here we show that only 5% of known imprinted genes with paternal allele silencing are monoallelic versus 56% with paternal allele expression—this cardiac-specific phenomenon seems unrelated to CHD. Further, compared with control subjects, CHD subjects have a significant burden of both LOE genes and ASE events associated with altered gene expression. These studies identify *FGFBP2*, *LBH*, *RBFOX2*, *SGSM1* and *ZBTB16* as candidate CHD genes because of significantly altered transcriptional expression.

## Abstract (web summary)

Congenital heart disease (CHD) is a disorder that occurs in ∼1% of live births. Here the authors describe a genome-wide allele-specific expression analyses in CHD patients, identifying five new genes involved in CHD and showing that paternally-expressed imprinted genes are monoallelic, while maternally-expressed imprinted genes are biallelic.

## Graphical abstract

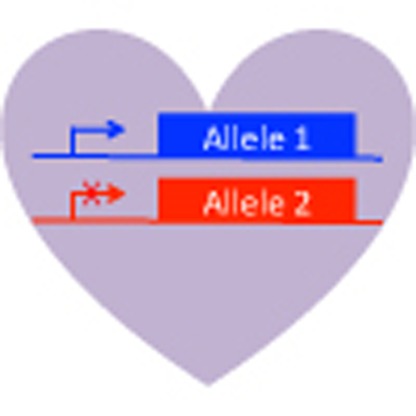

Congenital heart disease (CHD)-causing mutations have been identified in >50 genes including transcription factors, signalling molecules^1^,^2^,^3^ and chromatin modifiers^4^,^5^,^6^,^7^,^8^, which direct the temporal and spatial expression of genes during cardiac development. Recent studies have estimated that there are ∼400 genes that can harbour loss or gain-of-function mutations that cause CHD (denoted CHD genes)^4^,^8^. We hypothesized that other CHD genes could be identified by altered expression of one (allele-specific expression (ASE)) or both alleles (loss-of-expression (LOE); Fig. 1).

ASE occurs when transcription from one allele is selectively silenced or enhanced, or when transcripts undergo selective post-transcriptional degradation (for example, nonsense-mediated decay; NMD). ASE occurs physiologically to control dosage effects of chromosome X-encoded genes in females^9^ and to silence the maternal or paternal allele of imprinted genes^10^. Transcription of one allele can be suppressed by allele-specific chromatin marks^11^, long noncoding RNAs^12^ or gene regulatory element mutations^13^. Other ASE studies include all genes where one allele is expressed at a statistically higher level than the other allele, an approach that estimates hundreds of ASE events per tissue and thousands of ASE events per cell; however, this strategy likely results in significant overestimates of ASE-event rates^14^,^15^,^16^.

We studied ASE in discarded tissues from CHD patients, hypothesizing that ASE events likely to cause CHD should result in substantial allele bias in the expressed transcripts. Hence, we focused on genes that are expressed in fetal heart that (1) are normally biallelically expressed and (2) exhibit extreme ASE (that is, >86% expression of one allele relative to the other). Moreover, we suggest that ASE *per se* would not be enough to cause a disease phenotype, particularly if dosage compensation resulted in overall normal gene expression. Thus, we focused on extreme ASE events, either with significantly altered gene expression or in which a deleterious mutation was detected in the expressed allele (Fig. 1) as candidate CHD genes.

Biallelic LOE (caused by inadequate *trans*-acting factors, or combinations of gene regulatory mutations and/or NMD) can cause CHD by either dominant or recessive mechanisms. To identify LOE genes potentially responsible for CHD, we focused on genes (1) that are highly expressed (upper quartile of expressed genes), (2) with tightly regulated cardiovascular expression and (3) with significantly downregulated (>10-fold) expression.

We demonstrate that 24% of extreme ASE events in CHD subjects are associated with significantly altered levels of gene expression (compared with 0% of extreme ASE events in control subjects). We identify nine genes in CHD subjects that are functionally null—three due to ASE with a damaging mutation in the expressed allele and six due to biallelic LOE. We propose *FGFBP2*, *LBH*, *RBFOX2*, *SGSM1* and *ZBTB16* as especially strong candidate CHD genes.

## Results

### RNAseq expression analyses of CHD subjects and controls

To identify cardiac gene expression, we studied 144 probands (average age, 2.9 years; range, fetal to 21 years) enrolled in the Pediatric Cardiac Genomics Consortium^17^ and performed RNA sequencing (RNAseq) on 172 surgically discarded cardiovascular tissues (Supplementary Data 1 and 2). We also studied gene expression data from ‘normal' adult (average age, 49.3 years; range, 20–70 years) cardiac tissues (*n*=87, left ventricle; *n*=26, right atria) from the Genotype-Tissue Expression Consortium^18^ (GTEx, *n*=95 subjects; Supplementary Data 3 and 4) and fetal cardiac tissues without CHD (*n*=5, gestational age 15–16 weeks).

RNA expression was measured using standard RNAseq procedures (see Methods). The 172 CHD samples were obtained from eight different cardiovascular tissues (aorta, atrial septum, ductus arteriosus, interventricular septum, left ventricle, pulmonary artery, right atrium and right ventricle; Supplementary Data 2 and Supplementary Table 1) with at least six samples per tissue. Approximately 15,300 expressed genes were expressed in each sample (two or more aligned reads per million (r.p.m.); Fig. 2a (grey bars) and Supplementary Table 1), and the average number of genes expressed per subject per tissue ranged from 14,938 (interventricular septum) to 15,883 (pulmonary artery). Expression analyses were performed by comparing single samples to the mean of all other samples of the same tissue type (for example, right atrial CHD sample versus all other right atrial CHD samples).

### Identification of extreme ASE events

To detect extreme ASE events in genes that are normally biallelically expressed, single-nucleotide polymorphisms (SNPs) were identified from whole exome^4^,^8^ (WES, *n*=130) or genome (WGS, *n*=14) sequencing of CHD probands and available unaffected parents. SNPs were identified in GTEx donors from Illumina Exome and 5M SNP arrays. At each genomic heterozygous SNP (see Methods), an allele ratio (reference RNAseq reads/alternate RNAseq reads) was computed (Supplementary Fig. 1). To focus on ASE events in genes with normal biallelic expression, SNPs with typical biased expression were excluded (see Methods, Supplementary Fig. 1 and Supplementary Table 2). SNPs were then phased using parental genotypes or by assuming that the most highly expressed base at each SNP is encoded by the same allele. Compound allele ratios were then calculated for each gene as the ratio of the summed reads corresponding to each allele (Methods). This phasing methodology was 98% accurate in F1 mouse tissues (*M. musculus* × *M*. *castaneus*; Supplementary Table 3) and 100% accurate in three human WGS trios (affected proband and unaffected parents), when we applied a compound allele ratio threshold ≥7 (Supplementary Fig. 2c). Transcripts with a compound allele ratio ≥7.2 and a binomial *P* value<0.01 (Bonferroni-corrected for the number of expressed genes with heterozygous SNPs (Supplementary Data 2 and 4)) were designated as having extreme ASE. Finally, both over-represented ASE genes (that is, >5% of subjects including at least one control subject have ASE events in the same gene; Supplementary Table 4) and ASE events in genes with low fetal heart expression (Supplementary Table 5) were removed.

DNA-sequencing methodology strongly influenced extreme ASE detection (Fig. 2b). WGS and WES data yielded 5.1 and 1.4 extreme ASE events per CHD subject, respectively; SNP arrays yielded 3.3 extreme ASE events per GTEx donor. In total, we detected 607 extreme ASE events; 491 events in 17 known imprinted genes^19^ (Supplementary Data 5 and www.geneimprint.com) and 116 events (CHD, 56; GTEx, 60; Supplementary Tables 6 and 7) in genes normally biallelically expressed. Dideoxy sequencing of cDNAs from CHD tissues confirmed 17/17 ASE events in imprinted genes (100% validation rate), and 45/56 ASE events in non-imprinted genes (80% validation rate; Supplementary Figs 3 and 4 and Supplementary Data 6).

### Imprinting in cardiovascular tissues

Because parental gene imprinting is a major cause of ASE in mammalian tissues, we assessed imprinting in cardiovascular tissues. The set of genes imprinted in fetal and/or neonatal cardiovascular tissues has not been described. Forty-eight genes previously identified as being imprinted in non-cardiac tissues were expressed in fetal heart and also contained heterozygous SNPs. These were evaluated for ASE in cardiovascular tissues. Seventeen genes had ASE in at least 50% of CHD and GTEx subjects (Table 1), whereas 31 were predominantly biallelically expressed (Supplementary Table 8). Gene imprinting in mouse cardiovascular tissues was the same as in human cardiovascular tissues except for three genes: *CDKN1C*, *IGF2R* and *SGCE*. These three genes are biallelically expressed in human hearts, but have ASE in mouse hearts. Biallelic expression of these same genes in other human tissues has been described^20^,^21^,^22^. After excluding differences that were attributable to genotyping methods, ASE of imprinted genes was indistinguishable in CHD proband tissues and GTEx tissues. ASE events in known imprinted genes were excluded from further analyses.

### Extreme ASE is attributable to NMD in a minority of cases

We identified 78 rare, nonsense mutations in genes that were expressed at sufficient levels to evaluate ASE in CHD probands (Supplementary Table 9). Only 14/78 (18%) genes exhibited NMD and had significantly reduced expression of the allele harbouring the LOF mutation, even after employing a less stringent definition of ASE (allele bias >4). This low percentage of NMD is consistent with previous reports^23^. Unfortunately, this analysis could not be performed on GTEx samples because complete coding sequence data were unavailable for these subjects.

Of the 45 extreme ASE events observed in CHD probands (Supplementary Table 6), seven ASE events (Table 2) resulted from nonsense (*ASPN*, *CTSA, PGM1* and *RBFOX2*), splice site (*AARSD1*) or frameshift (*C7* and *RETSAT*) variants that caused NMD. In sum, 38/45 (85%) extreme ASE events in CHD probands remain unexplained and could potentially reflect mutations in gene regulatory elements.

### Extreme ASE genes have significant expression changes

We hypothesized that genes whose expression levels differ between subjects with extreme ASE versus subjects with biallelic expression are candidate CHD genes. After excluding eight CHD and three GTEx tissues that failed quality controls (Methods), we compared gene expression in 37 extreme ASE genes in CHD subjects and 57 extreme ASE genes in GTEx subjects to the mean expression of biallelically expressed samples of the same tissue type (for example, right atrial ASE expression compared with right atrial biallelic expression). In CHD subjects, upregulated ASE genes (fold >5, *P*<0.05, *P* calculated from *z*-score)) included *MYOZ1* and *FGFBP2* (observed in two subjects) and downregulated ASE genes (fold <0.65, *P*<0.05, *P* calculated from *z*-score) included *SGSM1*, *AARSD1*, *C5orf46*, *SDHB*, *CBR1* and *RBFOX2* (Table 3). By contrast, no extreme ASE gene had significantly different expression in GTEx subjects.

### Identification of functionally null genes

We identified CHD gene candidates who had extreme ASE and harboured a deleterious mutation in the expressed allele, and so are unlikely to make functional protein. Three extreme ASE genes, *C17orf97*, *CRACR2B* and *FGFBP2,* encoded rare, putatively deleterious variants in the expressed allele (Table 2). Although *FGFBP2* has relatively common ASE (>5% of subjects (Supplementary Table 6)), its functional null status makes it a candidate-recessive CHD gene. These analyses could not be performed in GTEx samples.

Biallelic LOE of genes that are typically both highly expressed and tightly regulated are another class of functionally null genes that may cause CHD. We identified genes with biallelic LOE (fold <0.1, *P*<2.7 × 10^−3^ (*z*-score<−3), *P* calculated from *z*-score) in six CHD probands (Table 3) but none in GTEx donors (*P*=7.8 × 10^−3^, Fisher Exact test). Five LOE genes, *LBH*, *FRG1B, PHKG1*, *IRX5* and *ZBTB16*, have no homozygous LOF variants in the Exome Aggregation Consortium (ExAC) database (exac.broadinstitute.org), while *TRMT2B*, an X-linked gene, is hemizygous in a significant number of subjects. Significant downregulation of all biallelic LOE genes was confirmed using quantitative PCR (qPCR; Supplementary Table 10), although this analysis identified only an approximately threefold reduction in *FRG1B* and *IRX5*. Biallelic LOE of *PHKG1* and *IRX5* occurred in two subjects both with Kabuki syndrome (MIM147920 (ref. 7)), caused by damaging *de novo KMT2D* mutations^4^.

## Discussion

Our transcriptome analyses of tissues from CHD patients identified several CHD gene candidates, including *RBFOX2,* a recently discovered definitive CHD gene^8^. Although two LOE genes, *FRG1B* and *TRMT2B,* have no known role in cardiac development, the known functions of other candidate genes increase the likelihood that altered expression could cause CHD. In addition, we identified two novel findings related to cardiovascular imprinted genes.

There were no significant differences in imprinting between CHD and GTEx subjects, suggesting that imprinting defects are not a common cause of CHD. However, we note the following unexpected observations. First, the imprinted gene *RTL1* has maternal ASE in both human and mouse cardiovascular tissues (Table 1 and Supplementary Table 11) but the opposite (paternal) allele is expressed in other fetal and placental tissues^24^,^25^. Temporal and spatial-dependent parental allele switching has been observed in only two other imprinted genes, *GRB10* and *IGF2* (refs 26, 27). Second, *RTL1* is the only protein-coding, imprinted gene with maternal ASE in cardiovascular tissues. Imprinted genes in other tissues do not have a significant maternal or paternal bias. In cardiac tissues, imprinted gene clusters were significantly biased (*P*=0.008, Fisher Exact test) for paternal ASE (11 ASE; 8 biallelic) versus maternal ASE (1 ASE; 12 biallelic). Only 5% (1/21) of maternally expressed, imprinted genes had ASE, while 56% of paternally expressed, imprinted genes (14/25) had ASE (*P*=3.1 × 10^−4^, Fisher Exact test). Together, these data suggest a cardiac-specific mechanism that removes imprinting ‘marks' that would normally silence maternal allele expression.

Although extreme ASE events were identified in both CHD and GTEx subjects, ASE associated with significantly altered gene expression was observed only in CHD subjects (9/37 CHD subjects; 0/57 GTEx subjects; *P*=1.1 × 10^−4^, Fisher Exact test). As genes with extreme ASE and downregulated expression could phenocopy genes harbouring loss-of-function (LOF) mutations in one allele (ignoring potential dominant-negative effects), we compared the LOF allele frequency (AF) between CHD and control data sets. Five of six ASE genes with reduced expression are constrained and have a low frequency (<0.001) of LOF mutations in the ExAC database (Table 3). Three of these, *AARSD1*, *SDHB* and *C5orf46*, have no known functions in the heart. The other two genes, *RBFOX2* and *SGSM1*, are strong candidates for contributing to CHD. Prior WES analyses identified *de novo RBFOX2* LOF variants in four CHD probands (including the subject with ASE), and *SGSM1* LOF variants in two CHD probands (one each *de novo* and inherited), but no *RBFOX2* or *SGSM1* LOF variant in 1,800 controls^3^,^4^,^8^. The LOF AF was significantly higher in CHD probands for both *RBFOX2* (CHD AF=1.4 × 10^−3^; ExAC AF=1.8 × 10^−5^; odds ratio (OR)=78; *P*=6.6 × 10^−5^, Fisher Exact test) and *SGSM1* (CHD AF=9.4 × 10^−4^; ExAC AF=9.1 × 10^−5^; OR=10.4; *P*=0.02, Fisher Exact test) than observed in ∼55,000 control exomes. Moreover, the estimated odds ratio for *SGSM1* is conservative as we excluded both the subjects with ASE and another CHD subject with markedly reduced biallelic expression (1-02922: 0.19-fold, *P*=0.02, *P* calculated from *z*-score).

*FGFBP2*, another strong CHD gene candidate, is predicted to lack all gene functions in two CHD subjects, through two different mechanisms. One subject had extreme ASE with a deleterious mutation in the expressed allele (Table 2), while the other subject is predicted to have complete loss of *FGFBP2* gene function due to severely reduced biallelic expression (1-02697, Table 3). Notably, *FGFBP2* is a component of the FGF signalling axis that regulates outflow tract and valve morphogenesis^28^, and both CHD subjects with abrogated *FGFBP2* expression have outflow tract defects.

Two probands with abnormal valve development had biallelic LOE of *LBH* (limb bud and heart) or *ZBTB16*. *LBH*, a cardiac developmental transcriptional co-activator, mediates neural crest migration^29^, which is required for aortic valve formation^30^. Overexpression of mouse *Lbh* produces valvular defects and decreases *Nppa* (atrial natriuretic hormone) expression^31^. Consistent with this, LOE of *LBH* in proband 1-03051 was associated with increased *NPPA* expression (fold=12.3, *P*<0.0001, *P* calculated from *z*-score). However, mice lacking *Lbh* have no overt cardiovascular defects^32^. *ZBTB16* (also known as *PLZF*) is a member of the Krueppel (C2H2-type) zinc-finger transcription factor family that regulates expression of *GATA4* (ref. 33); GATA transcription factors are important for outflow tract development^34^. *ZBTB16* LOE in proband 1-03316 was associated with reduced *GATA4* expression (0.04-fold, *P*=0.19, *P* calculated from *z*-score).

Kabuki syndrome is a complex developmental disorder including CHD caused by mutation in *KMT2D*, a histone methyltransferase. Our studies identified markedly reduced expression of *PHKG1* in two Kabuki syndrome subjects (Table 3) and decreased expression of *IRX5* in one subject. (*IRX5* expression is normally low in right atrial tissues, the only sample available from subject 1-00596.) Damaging *IRX5* mutations (MIM611174 (ref. 35)) cause CHD with conduction abnormalities, marked frontonasal anomalies and prominent ears, phenotypes that overlap that also occur in Kabuki syndrome. On the basis of these data, we speculate that *KMT2D* regulation of *IRX5* and *PHKG1* contributes to the pathogenesis of Kabuki syndrome.

Although we identified nine ASE events likely related to CHD in 144 probands, our analyses have several limitations. First, most of the CHD tissues were acquired after birth, and genes with aberrant expression that are developmentally downregulated would escape detection. Second, only ∼25% of cardiac expressed genes contain heterozygous SNPs per sample, so a large fraction of ASE genes cannot be detected by our methodology. We also detected 3.6-fold more ASE genes in subjects genotyped with WGS than subjects genotyped with WES. WES genotyping does not evaluate untranslated region (UTR) sequences and, hence, does not discover SNPs in these regions, resulting in low efficiency of ASE detection in exome-genotyped samples. Finally, to limit false-positives, we employed stringent definitions of ASE (≥7.2 allele ratio; *P*≤0.01, Bonferroni-corrected binomial distribution) and LOE (fold<0.1, *P*<2.7 × 10^−3^, *P* calculated from *z*-score) genes, at the expense of under-calling expression differences. We expect that cardiac RNA expression from more CHD tissues will explain a larger proportion of disease.

We found that only a small subset of extreme ASE events was attributable to NMD. Hence, unknown mechanisms accounted for allele gain/loss of gene expression in the majority (85%) of extreme ASE events identified in CHD subjects. While we speculate that mutations in regulatory sequences may lead to altered allele-specific transcription, another contributing factor could be somatic mutations expressed in cardiovascular tissues, but not blood (the DNA source for genomic sequencing), that cause NMD.

In summary, integrated analyses of genomic DNA and RNAseq in CHD cardiac tissues identified preferential silencing of paternally expressed imprinted genes, several extreme ASE and LOE genes relevant to cardiogenesis and potential downstream targets of *KMT2D*. DNA sequence analyses of 81 trios identified nine *de novo* mutations likely responsible for disease^4^,^8^. Assessment of RNAseq data from these and 63 singletons identified seven instances (*RBFOX2*, *SGSM1* (*n*=2), *FGFBP2* (*n*=2), *LBH* and *ZBTB16*) with significantly reduced gene expression likely contributing to CHD. These data support the use of RNAseq analyses in identifying disease genes. We expect that further study of WGS will identify damaging mutations in regulatory elements that alter transcription of these CHD genes.

## Methods

### Patient and control cohorts

CHD probands were recruited from nine centres in the United States and the United Kingdom into the Congenital Heart Disease Genetic Network Study of the Pediatric Cardiac Genomics Consortium (CHD Genes: NCT01196182). The protocol was approved by the Institutional Review Boards of Boston Children's Hospital, Brigham and Women's Hospital, Great Ormond St Hospital, Children's Hospital of Los Angeles, Children's Hospital of Philadelphia, Columbia University Medical Center, Icahn School of Medicine at Mount Sinai, University of Rochester School of Medicine and Dentistry, Steven and Alexandra Cohen Children's Medical Center of New York and Yale School of Medicine. Written informed consent was obtained from each participating subject or parent/guardian. Probands with CHD were selected based on availability of cardiovascular tissue and RNA quality (RNA integrity number, RIN). Cardiac diagnoses were obtained from review of echocardiogram, catheterization and operative reports; extracardiac findings were extracted from medical records.

The control cohort consisted of RNAseq data from 113 heart tissues (left ventricle and/or right atrium) from 95 deceased subjects who were enroled in the GTEx programme. The GTEx data sets used for the analyses described in this manuscript were obtained from: dbGaP through dbGaP accession number phs000424.vN.pN on 02 August 2014.

### WES and WGS

Exomes of CHD probands were captured and sequenced at the Yale Center for Genome Analysis, as described^4^. In brief, gDNA isolated from venous blood was captured with the NimbleGen v2.0 exome capture reagent (Roche) and sequenced (Illumina HiSeq 2000, 75 base paired-end reads) to a mean read depth of 107. Reads were aligned to the hg19 reference genome using Novoalign (Novocraft), and variants called using HaplotypeCaller (Genotype Analysis Toolkit, GATK)^36^. Variants were filtered using the hard filters (FisherStrand (FS)<25, quality by depth (QD)<4) for passing variants. Identified heterozygous SNPs had a minimum genotype quality score of 50 and an allele balance (AB, number ALT reads/(number REF reads+number ALT reads), where ‘ALT' and ‘REF' reads refers to reads containing the alternate or reference base in a heterozygous SNP) between 0.2 and 0.8.

Whole genomes of 11 probands and three trios were sequenced to an average read depth of 35.2. gDNA libraries were made from 5 μg of purified DNA and sequenced on an Illumina HiSeq 2000 (101 base paired-end reads). Reads were aligned to reference genome hg19 using Novoalign (Novocraft) and variants were called using UnifiedGenotyper and filtered by VQSR (GATK^36^). Heterozygous SNPs were identified using the same criteria as for WES.

### SNP array genotyping

GTEx subjects were genotyped on both Illumina Exome and Illumina 5M arrays.

### Variant annotation and minor allele frequency

Variants were annotated using SNPEff^37^. Damaging missense variants were predicted using both Polyphen2 (ref. 5) and CADD^38^. Minor AF (MAF) information for each SNP was extracted from the ExAC database, containing >55,000 individuals. If AF data were unavailable from ExAC, the maximum MAF reported in dbSNP, Exome Variant Server, HapMap or 1000 Genomes was chosen for subsequent calculations.

### RNAseq and analyses

RNA was purified from RNAlater-treated frozen tissue, using Trizol (Life Technologies). RNA (RIN>5) was converted into cDNA and into RNAseq libraries as described^39^. In brief, purified poly-A RNA that had gone through two rounds of oligo-dT selection was converted into cDNA and then made into RNAseq libraries. Libraries were sequenced (Illumina HiSeq 2000 or Illumina HiSeq 2500, 50-base paired-end reads) to a target depth of >20 million reads (median, 57 million reads; range, 20–530 million reads). Reads were aligned to the hg19 reference genome using TopHat 1.4 (using the following parameters: ‘-m 1 -a 5 --segment-mismatches 3 --segment-length 25 -g 0 --no-novel-juncs', with splice junctions being defined by genes.gtf (Illumina iGenome download)). Mitochondrial and duplicate reads were discarded using Samtools and Picard's MarkDuplicates, respectively. A median of 60% of reads was aligned to the reference genome, hg19, and 36% of reads uniquely aligned to the nuclear genome. Allele-specific reads were tallied using GATK UnifiedGenotyper at each heterozygous position identified by gDNA sequencing (using the following parameters: ‘--genotyping_mode GENOTYPE_GIVEN_ALLELES --alleles het_snps_only.vcf --output_mode EMIT_ALL_SITES' where a personalized vcf file containing only heterozygous SNP was used as ‘het_snps_only.vcf'). Gene expression was determined by calculating reads per gene per million aligned reads (r.p.m.).

### Quality-control metrics for subjects

Some subjects were excluded from our study. Ignoring SNPs with expected ASE (chromosome X genes, previously reported imprinted genes, and SNPs within alternatively spliced exons), we observed that most heterozygous SNPs (with a minimum of 10 reads) were expressed biallelically (CHD exome (95.7±1.0%), CHD WGS (96.8±0.5%) and GTEx (97.6±0.6%; Supplementary Fig. 2a). Eight CHD and three GTEx subjects with substantially lower biallelic SNP expression (<75%; data not shown) were removed from this study.

### Quality-control metrics for SNPs

To ensure accurate ASE identification, all genotyped SNPs observed in RNAseq data were subjected to quality control. Only SNPs with at least five reads in RNAseq data or both alleles expressed were analysed for ASE.

#### Low-quality genotype called SNPs

Low-quality SNPs observed in either WES or WGS generally reflected either misaligned DNA sequence reads because of gene orthologues, pseudogenes or other highly similar sequences. As previously described, low-quality SNPs either failed GATK variant filtration or had significantly biased AB. In addition, genes with a single heterozygous SNP, expressed at a high level (>20 reads), with 100% monoallelic expression were excluded; the reference base of these SNPs was expressed in >80% of cases (Supplementary Fig. 2b), indicating a suspicious genotype.

#### SNPs in alternatively spliced exons

Biased expression of SNPs in alternate exons could not be evaluated for ASE because we could not differentiate allele-specific expression versus allele-specific splicing. That is, SNPs (Supplementary Data 7, annotated ‘Filter_SNP_alt_splicing') in exons that were only found in a subset of gene isoforms, as represented in three databases (RefGENE.txt (Illumina), UCSC Genes and Basic Gene Annotations Set (ENCODE/GENCODE)), were excluded.

#### SNPs with suspected alignment biases

Sequencing reads containing clustered SNPs (that is, those within 30 bp of two other SNPs) or SNPs in close proximity to an indel (within 30 bp of an indel) were often not aligned by TopHat/Bowtie introducing artefacts that appear as allele biases; these SNPs were excluded. In addition, SNPs in repeat regions (Supplementary Data 7, annotated ‘Filter_SNP_duplicate_sequence') were excluded if SNP and flanking sequences (50nt up- and downstream) aligned to multiple genomic locations (identified with BLAT (UCSC Genome Browser)) and if the ALT base was the REF base at one of those multiple locations.

#### SNPs biased in multiple subjects

Some ‘common' biased SNPs (biased in >40% of CHD probands or GTEx subjects, with a minimum of three biased subjects) were present in genes with biallelically expressed SNPs. These common biased SNPs (except those likely to contribute to NMD) were filtered out as either not likely to impair cardiac development or as technical artifacts.

### Quality-control metrics for genes

#### Genes expressed in an allele-specific manner in many subjects

Excluded genes included all chromosomes X and Y genes, HLA- genes (that is, HLA-A, HLA-B, HLA-C, HLA-DMA, HLA-DMB, HLA-DOA, HLA-DOB, HLA-DPA1, HLA-DPB1, HLA-DQA1, HLA-DQA2, HLA-DQB1, HLA-DQB2, HLA-DRA, HLA-DRB1, HLA-DRB5, HLA-E, HLA-F, HLA-G, HLA-J, HLA-P and HLA-T) and noncoding genes. Coding genes were identified by SNPEFF_EFFECT designations: CODON_CHANGE_PLUS_CODON_DELETION, CODON_CHANGE_PLUS_CODON_INSERTION, CODON_DELETION, CODON_INSERTION, FRAME_SHIFT, NON_SYNONYMOUS_CODING, START_GAINED, STOP_GAINED, SYNONYMOUS_CODING, UTR_3_PRIME, UTR_5_PRIME. In addition, genes with ASE in >5% of subjects, including at least one GTEx subject, are unlikely to impair cardiac development; these ‘common' ASE genes are reported in Supplementary Table 4.

#### RNAs with misaligned reads

Heterozygous SNPs were excluded if >20% of any other heterozygous SNPs in the same transcript were not expressed, or if a heterozygous coding SNP was not expressed. This pattern reflected misaligned reads.

#### Genes with low fetal heart expression

RNAs that are unlikely to be involved in cardiac development (normalized fetal heart expression <2 r.p.m.) were filtered out (Supplementary Table 5). This filter removed five and 24 ASE events from CHD probands and GTEx donors, respectively.

### Quality-control confirmation of extreme ASE

#### Allele bias observed in both aligned and unaligned reads (that is fastq files)

As noted above, TopHat/Bowtie alignment can introduce apparent allele bias into aligned RNAseq data. To confirm allele bias was not introduced by TopHat/Bowtie alignment, raw, unaligned sequencing reads containing: 10nt flanking the ALT/REF base, or 20nt either upstream or downstream of the ALT/REF base, or their reverse complements, were counted. The numbers of raw sequencing reads containing ALT and REF sequences were required to be similar to the numbers of ALT and REF aligned reads. Further, allele balances were required to be similar in both unaligned and aligned read counts.

#### Visual inspection of RNAseq data in Integrative Genomics Viewer (Broad Institute)

Biased SNPs (Supplementary Data 7) were filtered out if other SNPs with biallelic expression were observed in the same gene—this was particularly important for CHD exome samples, which are not well genotyped in the 3′ UTR and for GTEx samples (in which genotyping was restricted to common SNPs). In addition, biased SNPs were excluded if visual inspection identified more than two alleles. Thirty-nine SNPs (Supplementary Data 7: ‘Filter_SNP_complex_allele_structure') had multiple alleles likely due to misalignment of reads from pseudogenes and/or gene families.

#### Confirmation of ASE events by Sanger sequencing

At least one biased SNP per extreme ASE event was analysed using Sanger sequencing. PCR products derived from RNA were prepared from 200 ng total RNA by incubation with Superscript III Reverse Transcriptase (Thermo Fisher) and then cDNA was PCR-amplified with Phusion polymerase (New England Biolabs) using gene-specific primers (Supplementary Data 6) flanking the SNPs of interest. PCR products were gel-purified using the QIAquick Gel Extraction Kit (QIAgen) and Sanger sequenced (GENEWIZ, Boston). The relative peak heights of the ALT and REF alleles were measured. Extreme ASE events were confirmed when the relative peak height was >5.

### Quality controls removed REF allele bias

Unless the ALT base causes NMD, there should be no REF base versus ALT base expression bias in monoallelic SNPs. Before removal of ‘low-quality' genome-wide SNPs, 87.1% of ASE events express the REF base, whereas after quality control, ∼50% of biased SNPs express the REF base (Supplementary Fig. 2b).

### Allele bias and ASE *P* value calculation

Allele bias and ASE *P* value were calculated for each SNP that passed quality-control measures. If there were multiple SNPs per gene, we either used phasing of SNPs (from maternal (mat) and paternal (pat) alleles) or we made the assumption that if there are multiple SNPs in a given gene, the expression bias will be unidirectional; that is, polymorphic bases with higher expression are on the same allele. Allele bias was calculated as follows:

Reads containing heterozygous SNPs were counted and binned into one of four categories, based on inheritance and expression: (1) maternal inheritance, (2) paternal inheritance, (3) unknown inheritance with higher-allele expression and (4) unknown inheritance with lower-allele expression. For each heterozygous SNP in a gene, reads were summed into one of four allele categories (1) SNP-mat sum, (2) SNP-pat sum, (3) SNP-higher-allele sum and (4) SNP-lower-allele sum. These four allele categories were reduced to two, as follows:

If allele inheritance can be determined:

If SNP-mat sum>SNP-pat sum

Allele bias=(SNP-higher-allele sum+SNP-mat sum)/(SNP-lower-allele sum+SNP-pat sum)

If SNP-pat sum>SNP-mat sum

Allele bias=(SNP-higher-allele sum+SNP-pat sum)/(SNP-lower-allele sum+SNP-mat sum)

If allele inheritance is unknown:

Allele bias=SNP-higher-allele sum/SNP-lower-allele sum

ASE *P* value is calculated using a binomial distribution model, and Bonferroni-corrected by the number of genes containing expressed heterozygous SNPs for each sample (Supplementary Data 2 and 4).

The assumption that the more highly expressed bases at heterozygous SNP positions are all on the same allele has the potential to introduce error into allele bias and statistical assessment of ASE. We directly tested this approach by studying F1 crosses of wild-type C57Bl6 and Castaneus mice. On the basis of parental mouse strain germline DNA sequence (Mouse Genomes Project; Wellcome Trust Sanger Institute), we estimated that F1 mice would have 18.5 million heterozygous SNPs, encoded within 19,236 transcripts. To make this more comparable to human WGS data, we only assessed every 18th heterozygous SNP. From RNAseq libraries prepared from left and right atrium, left and right ventricle, pulmonary artery, liver and skeletal muscle from P1 mice, we identified, on average, 14,678 expressed genes including 9,312 expressed genes with heterozygous SNPs (Fig. 2a). We then determined the false-positive rate of assigning unphased SNPs to alleles by comparing fully phased SNPs to unphased SNPs while varying the minimum allele bias (4 to 7-fold) and minimum read depth of monoallelic SNPs (4, 5, … 10) in each condition (Supplementary Fig. 2c). We determined that an allele bias of 7.2, with a minimum depth of five reads, yielded one false ASE event and 62 ‘true' ASE events (1.59% false-positive rate). The false-positive rate associated with assigning SNPs to alleles was also assessed in three human WGS trios, using an allele bias of 7.2 and a minimum read depth of five. Fourteen ASE genes were called regardless of whether phasing was used to assign alleles, or whether phasing was ignored.

### Imprinted genes

Human genes previously identified as ‘imprinted' in any tissue were identified in either of two online databases (http://www.geneimprint.com and www.otago.ac.nz/IGC). Genes with very low human fetal heart expression (<2 r.p.m.) were excluded because they were unlikely to contribute to normal heart development. Allele bias was calculated for each subject with heterozygous SNPs, and ASE events identified as described above.

### NMD analyses

NMD was assessed as described previously^23^. That is, the allele carrying the LOF mutation was at least fourfold less than the normal allele and read-depth sufficient to suggest a statistically significant (*P*<0.01) difference in allele ratio. *P* values were Bonferroni-corrected for the number of heterozygous nonsense mutations per subject (and not the number of expressed genes with heterozygous SNPs), so many more ASE events associated with NMD were identified than in our global ASE analyses.

### Expression analyses

**Figure 1:**
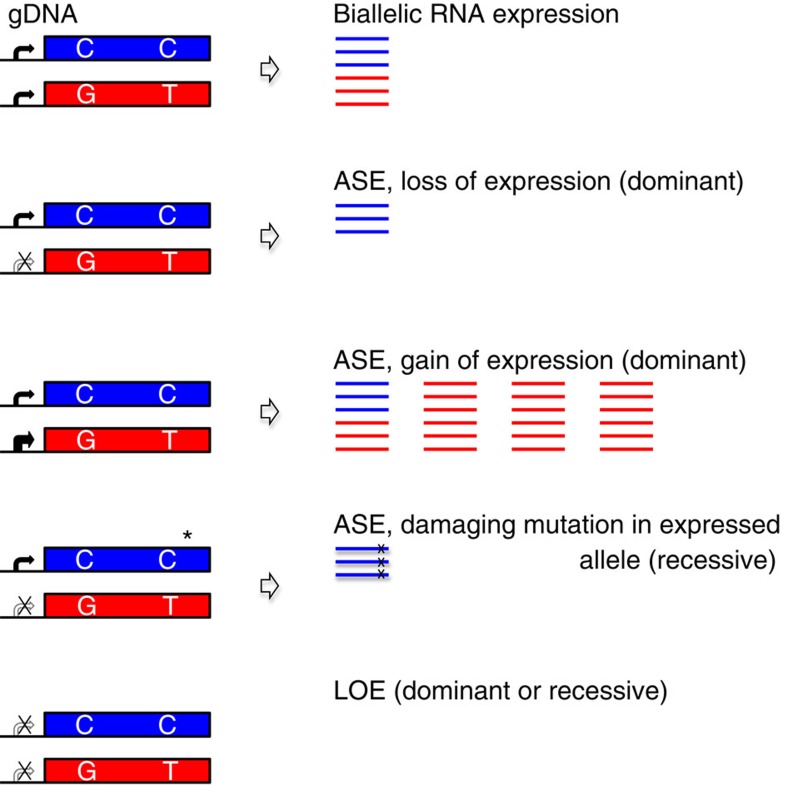
Identification of extreme ASE genes in subjects with CHD. Shown are both alleles of a gene that differ by the SNP haploblocks ‘CC' (blue) and ‘GT' (red), as identified by WES, WGS or SNP-array genotyping. RNAseq analysis (read counts at heterozygous positions) reveals the expression of both alleles (biallelic RNA expression) or the disproportionate expression of one allele over another (ASE). RNAseq expression analyses (comparing each sample to the average of all other samples within a tissue group) identify relative loss and gain of expression. Variant analysis, in conjunction with RNAseq analysis, can further identify LOF mutations in the expressed allele (*).

**Figure 2:**
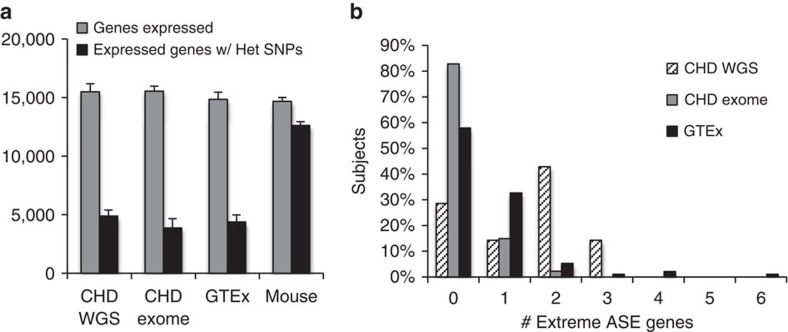
Extreme ASE genes preferentially identified in WGS subjects. Shown in **a** are the number of genes with a minimum expression of 2 r.p.m. (grey bars), and the number of expressed genes that contain heterozygous SNPs (black bars) for CHD WGS (*n*=30 tissues) and CHD WES probands (*n*=142 tissues), GTEx donors (*n*=113 tissues) and mouse C57Bl6/Castaneus F1 hybrids (*n*=7 tissues). s.d. is indicated. **b** The distribution of extreme ASE events per subject by genotyping platform. Extreme ASE events were identified in >70% of WGS subjects (*n*=14). However, extreme ASE events were identified in only ∼20% of WES subjects (*n*=130) and ∼45% of GTEx donors (*n*=95).

We calculated both r.p.m. and reads per million aligned reads per kilobase of transcript per gene per sample. On average, 15,479 genes (range, 14,938–17,651) were expressed in cardiovascular tissues (≥2 r.p.m.; Fig. 1b and Supplementary Table 1). Each sample was compared with the average expression of all other samples of the same tissue type (fold change), and statistical significance was assessed by *z*-score. As quality control, tissue groups with more than four samples were included in the analyses. Samples with >100 highly significant expression differences (fold<0.2, *P*<0.05 or fold >5, *P*<0.05, *P* calculated from *z*-score) were excluded from the analysis.

RNA expression of ASE genes, which demonstrated significant downregulation (fold<0.65, *P*<0.05) or significant upregulation (fold >5, *P*<0.05), were based on the expected fold change of ∼0.5 (allele loss of expression) and >7.2 (allele gain of expression), and relaxed by 30% because of the variability in gender, age and genotypes.

Fold downregulated: 0.5+(0.5 × 0.3)=0.65.

Fold upregulated: 7.2−(7.2 × 0.3)=5.

For LOE analyses, we required a more stringent definition of significant downregulation (fold<0.1, *P*<2.7 × 10^−3^, *P* calculated from *z*-score). This *P* value is based on a *z*-score<−3.

Reported LOE events (Table 3) are limited to polyadenylated transcripts because RNAseq libraries were constructed from polyA-selected mRNA.

### Quantitative RT–PCR

LOE events in CHD subjects, detected by RNAseq analyses, were confirmed using qPCR. cDNA was prepared from 200 ng RNA from the CHD proband and at least two matched tissue samples using Superscript III Reverse Transcriptase (Thermo-Fisher). cDNA was PCR-amplified using Phusion polymerase (New England Biolabs) with SYBR green and gene-specific primers (Supplementary Table 12) and analysed using Fast Real-Time PCR (Applied Biosystems). Housekeeping gene (*ACTB*, *GAPDH*, *GUSB* and *PPIA*) expression was used to calculate ΔCt, and ΔΔCt and *P* value (Student's *t*-test) are reported in Supplementary Table 10.

### Data availability

Clinical and sequence data that support the findings of this study have been deposited in dbGaP under accession phs000571. The data that support the findings of this study are available from the corresponding author upon request.

## Additional information

**How to cite this article:** McKean, D. M. *et al*. Loss of RNA expression and allele-specific expression associated with congenital heart disease. *Nat. Commun.* 7:12824 doi: 10.1038/ncomms12824 (2016).

## Supplementary Material

Supplementary Information Supplementary Figure 1-4 and Supplementary Table 1-12

Supplementary Dataset 1 CHD Subject Data

Supplementary Dataset 2 CHD RNAseq Data

Supplementary Dataset 3 Control Subject Data

Supplementary Dataset 4 Control RNAseq Data

Supplementary Dataset 5 Expression of genes that are imprinted in at least one human tissue

Supplementary Dataset 6 Sanger sequencing confirmation of ASE

Supplementary Dataset 7 Filtered Heterozygous SNPs

## Figures and Tables

**Table 1:** ASE of imprinted genes in cardiovascular tissues.

**Gene**	**Chr:Pos (hg19)**	CHD ASE*	GTEx ASE*	**% ASE**	**Coding/noncoding**	**Expressed allele**	**FHE**	**Mouse**
*ZDBF2*	chr2:207139522-207179148	4/7	15/15	86	C	P	8.2	ASE†
*NAP1L5*	chr4:89617065-89619023	9/10	14/14	96	C	P	18.3	NE
*FAM50B*	chr6:3849631-3851551	4/5	22/23	93	C	P	37.9	NE
*PLAGL1*	chr6:144261436-144385735	16/16	33/34	98	C	P	39.7	ASE
*PEG10*	chr7:94285636-94299006	1/1‡	0/0	100	C	P	98.8	ASE
*MEST*	chr7:130126015-130146138	7/8	1/1	89	C	P	47.7	ASE
*H19IGF2*	chr11:2016405-2170833chr11:2016405-2170833	12/124/4	68/691/1	99100	NCC	MP	32872677	ASEASE
*DLK1MEG3RTL1*	chr14:101193201-101373305chr14:101193201-101373305chr14:101193201-101373305	17/175/51/1‡	39/402/80/0	9854100	CNCC	PMM	76.4285.33.2	ASEASEASE†
*MAGEL2NDNSNRPNSNURF*	chr15:23888695-25244225chr15:23888695-25244225chr15:23888695-25244225chr15:23888695-25244225	2/255/5560/600/0	0/0NS48/484/4	100100100100	CCCC	PPPP	3.963.868.999.1	ASE†§ASEASEASE
*PEG3*	chr19:57321444-57352094	14/15	14/17	88	C	P	103.2	ASE
*NNAT*	chr20:36149606-36152090	1/2	0/0	50	C	P	21.1	NS

ASE, allele-specific expression; CHD, congenital heart disease; FHE, fetal heart expression (reads per million aligned reads); GTEx, Genotype-Tissue Expression Consortium; M, maternal; NE, not expressed; NS, no SNP; P, paternal; SNP, single-nucleotide polymorphism.

^*^Number subjects with silenced allele/number of informative subjects.

^†^Mouse ASE observed in pulmonary artery.

^‡^*PEG10* and *RTL1* are not expressed in postnatal cardiac tissues and are silenced in the only fetal subject in the study.

^§^Mouse ASE observed in skeletal muscle.

**Table 2:**
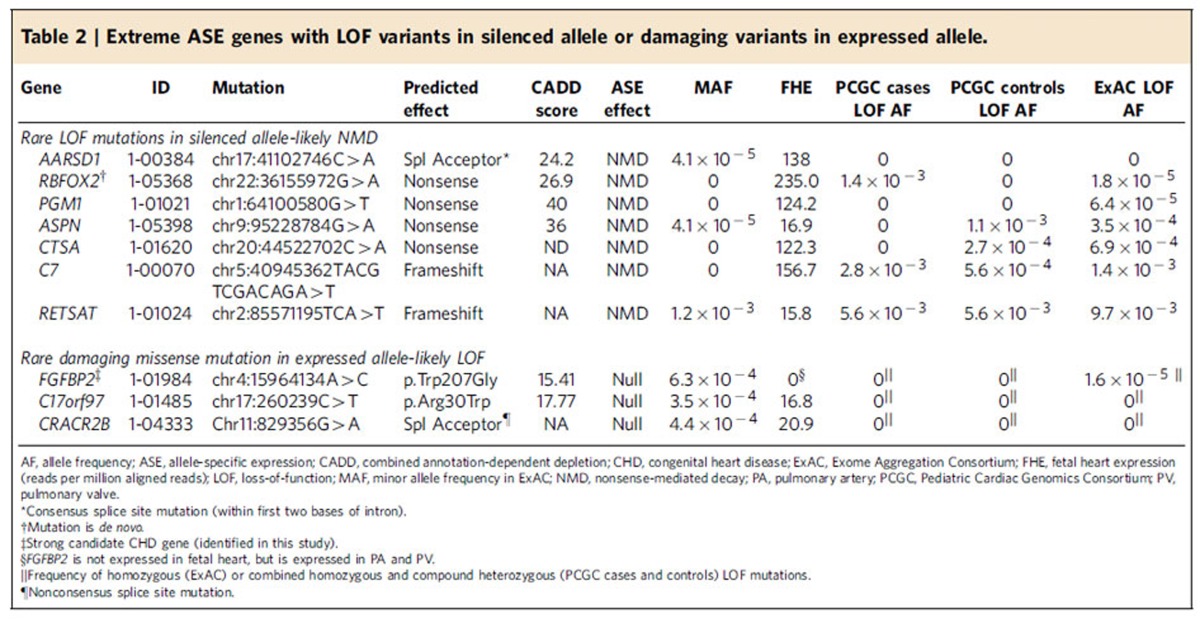
Extreme ASE genes with LOF variants in silenced allele or damaging variants in expressed allele.

*Reaction conditions:**1**/LiHMDS/**2**/[Pd(*η*^3^-C_3_H_5_)Cl]_2_/S-IPr·HCl=200/200/100/2.5/5; 0.1 M of ketone **1**; T=30^o^C; B/L and *dr* was determined by ^1^H NMR, *dr* is the ratio of (±)-(*syn,anti*)-**3**/other diastereoisomers; Isolated yield. †T=50 ^o^C. ‡Solvent=THF. §OBoc of **2** was replaced with OP(OEt)_2_. ||The yield was determined by ^1^H NMR.

**Table 3:** Extreme ASE and biallelic LOE events with significantly altered gene expression.

**Gene**	**ID**	**Tissue**	Proband Expr*	**Mean Expr ±s.d. (*****n*****)**	**Fold**	***P*** **value**†	**PCGC cases LOF AF**	**PCGC controls LOF AF**	**ExAC LOF AF**
*ASE genes with loss of allele expression*									
* RBFOX2*‡§	1-05368	DuctArt	189.1	296±44 (15)	0.64	1.6 × 10^−2^	1.4 × 10^−3^	0	1.8 × 10^−5^
* SGSM1*‡§	1-01019	RA	60.3	133±51 (18)	0.45	4.7 × 10^−2^	9.4 × 10^−4^	0	9.1 × 10^−5^
* AARSD1*	1-00384	IVS	65.9	108±12 (7)	0.61	3.0 × 10^−4^	0	0	0
* C5orf46*	1-00713	LV	7.7	42±17 (9)	0.18	4.4 × 10^−2^	0	0	9.1 × 10^−5^
* SDHB*	C417-01C417-01	IVSLV	169.6124.0	302±25 (7)224±46 (9)	0.560.55	<1.0 × 10^−4^3.0 × 10^−2^	0	0	8.2 × 10^−5^
* CBR1*	CHD-1548CHD-1548CHD-1548	LALVRA	12.915.110.6	29±6.0 (10)25±3.0 (7)26±3.4 (9)	0.440.600.41	6.1 × 10^−3^8.0 × 10^−4^<1.0 × 10^−4^	0	2.8 × 10^−4^	2.6 × 10^−3^
										
*ASE genes with gain of allele expression*										
* FGFBP2*	1-01024	RA	5.6	1.0±1.7 (15)	5.48	6.7 × 10^−3^	9.5 × 10^−4^	5.6 × 10^−4^	4.5 × 10^−4^	
* FGFBP2*	1-01984	LA	9.2	1.4±0.4 (3)	6.86	<1.0 × 10^−4^	9.5 × 10^−4^	5.6 × 10^−4^	4.5 × 10^−4^	
* MYOZ1*	1-02697	RV	26.9	4.2±3.5 (16)	6.45	<1.0 × 10^−4^	0	2.7 × 10^−4^	3.5 × 10^−4^	
										
*Genes with loss of expression of both alleles*										
* LBH*§	1-03051	AO	5.4	55±15 (6)	0.10	8.0 × 10^−4^	0||	0||	0||	
* ZBTB16*§	1-03316	AO	1.2	22±5.8 (6)	0.06	3.0 × 10^−4^	0||	0||	0||	
* IRX5*	1-03948¶	RV	0.6	13±4.1 (20)	0.05	1.9 × 10^−3^	0||	0||	0||	
* PHKG1*	1-00596¶	RA	2.6	30±8.7 (47)	0.09	1.5 × 10^−3^	0||	0||	1.6 × 10^−5^||	
* FRG1B*	1-04119	PA	0.3	5.4±1.1 (10)	0.06	<1.0 × 10^−4^	0||	0||	0||	
* TRMT2B*	1-02921	RV	0.0	5.5±1.3 (20)	0	<1.0 × 10^−4^	0||	0||	2.6 × 10^−4^||	
* PHKG1*#	1-03948¶	RV	0.6	15±8.1 (20)	0.04	4.0 × 10^−2^	0||	0||	1.6 × 10^−5^||	
* FGFBP2*#§	1-02697	PV	0.5	14±3.4 (3)	0.04	1.0 × 10^−4^	0||	0||	1.6 × 10^−5^||	

AF, allele frequency; AO, aorta; ASE, allele-specific expression; CHD, congenital heart disease; DuctArt, ductus arteriosus; ExAC, Exome Aggregation Consortium; IVS, interventricular septum; LA, left atrium; LOE, loss-of-expression; LOF, loss-of-function; LV, left ventricle; PA, pulmonary artery; PCGC, Pediatric Cardiac Genomics Consortium; PV, pulmonary valve; RA, right atrium; RV, right ventricle.

^*^Expression in reads per million aligned reads.

^†^*P* value calculated from *z*-score.

^‡^Includes *de novo* LOF mutations.

^§^Strong candidate CHD genes (identified in this study).

^||^Frequency of homozygous (ExAC) or combined homozygous and compound heterozygous (PCGC cases and controls) LOF mutations.

^¶^*KMT2D de novo* mutations identified in both 1-03948 (LOF) and 1-00596 (damaging-missense).

^#^LOE genes *PHKG1* (1-03948, *P*>2.7 × 10^−3^) and *FGFBP2* (1-02697, *n*=3) are not significant on their own, but occur in genes significant in other subjects.
