# Supplementary material for: Loss of RNA expression and allele-specific expression associated with congenital heart disease
Source: Nat Commun. 2016 Sep 27;7:12824. doi: 10.1038/ncomms12824 (PMC5052634; doi:10.1038/ncomms12824)
Supplement: Supplementary Information — Supplementary Figure 1-4 and Supplementary Table 1-12 [file ncomms12824-s1.pdf]

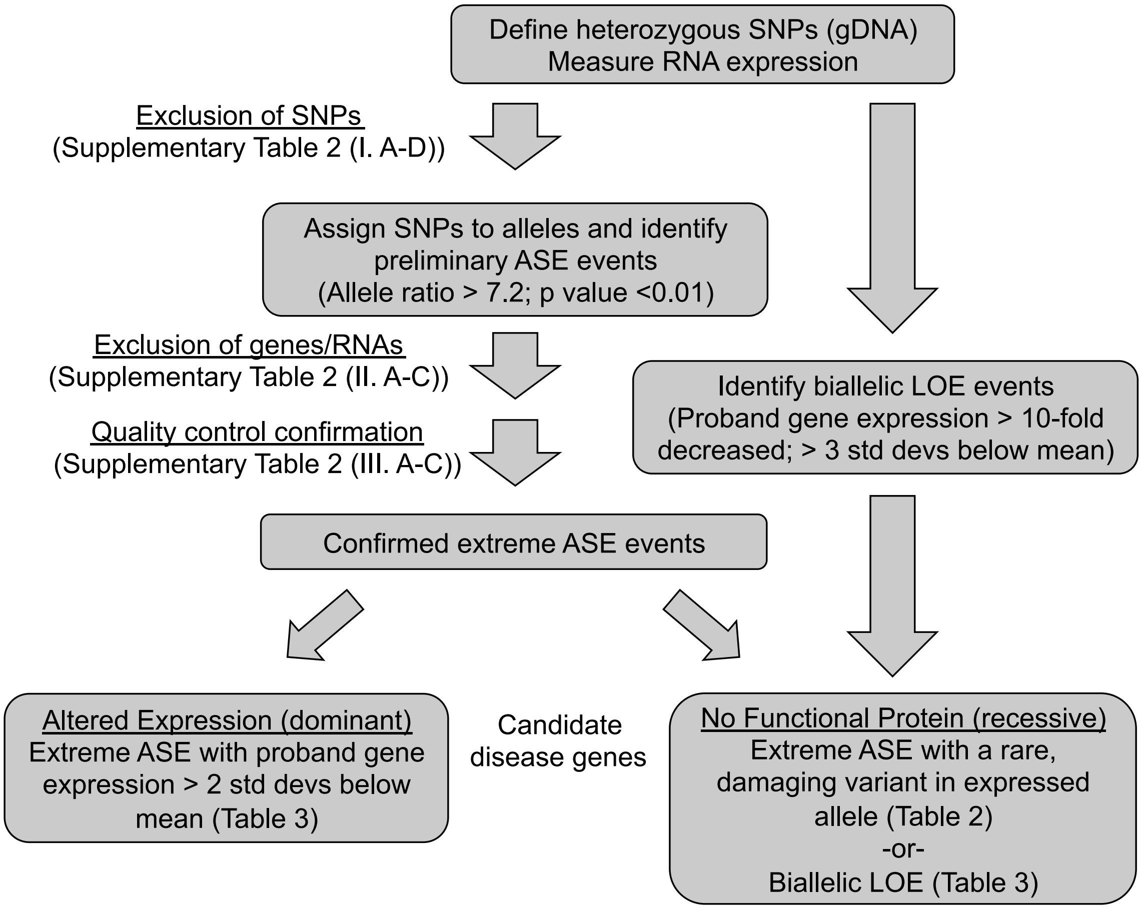

Supplementary Figure 1: Extreme ASE calling flowchart

RNAseq read counts were tallied at all heterozygous SNPs identified in the genomic DNA. Extensive SNP filtering was performed to exclude low quality SNPs, SNPs in alternatively spliced exons, SNPs with expected alignment biases, and SNPs that are repeatedly biased in multiple subjects. SNPs that passed these filters were then assigned to alleles, and a compound allele ratio and ASE p value was calculated for each gene. Preliminarily called ASE events (compound allele ratio > 7.2; ASE p value < 0.01; Bonferroni corrected binomial distribution) were then filtered to exclude noncoding genes, genes typically associated with ASE (ChrX genes, HLA- genes, known imprinted genes, and genes with ASE in >5% of subjects including at least one control subject (Supplementary Table 4)), genes harboring misaligned reads and genes with low fetal heart expression (Supplementary Table 5). Extreme ASE genes were then subjected to quality controls (manual inspection using the Integrated Genomic Viewer, confirmation that ASE exists in the fastq (unaligned) sequencing reads, and independent confirmation by Sanger sequencing. Filters used to exclude SNPs and genes/RNAs, as well as quality control confirmation are described in detail in Methods, and Supplementary Table 2 reports the number of SNPs and ASE events that were excluded per filter per subject. ASE genes with significantly altered expression or those that had a rare damaging variant in the expressed allele were deemed candidate disease genes.

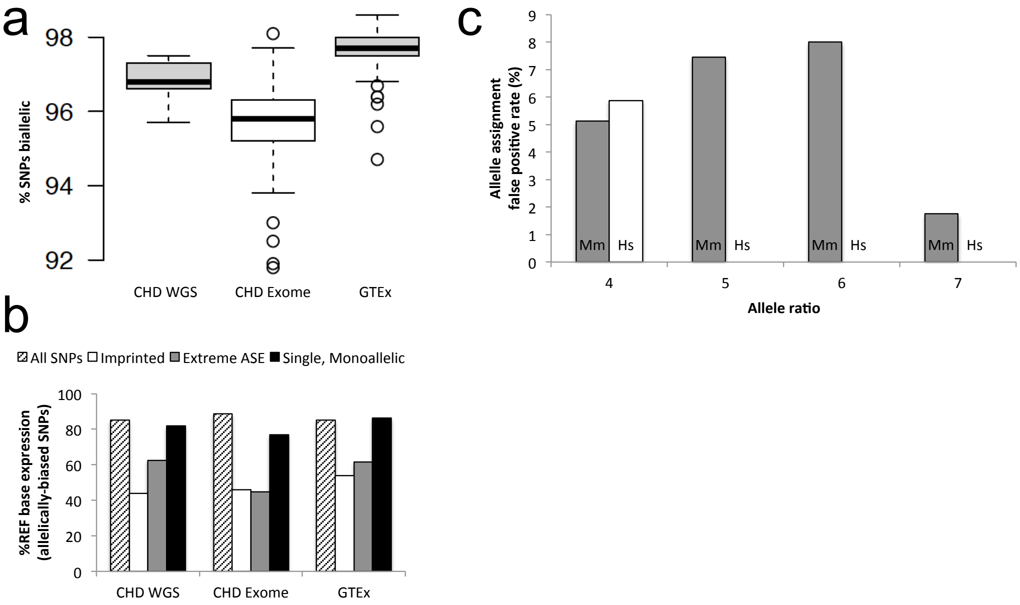

Supplementary Figure 2: Quality control of ASE calling algorithm
Genotyping and RNAseq data is highly concordant (a) as measured by determining the percent of heterozygous SNP positions that have biallelic expression (excluding SNPs with fewer than 10 RNAseq reads, SNPs on chromosome X, SNPs in alternatively spliced exons and SNPs in known or predicted imprinted genes). Box plots indicate the percent of SNPs with biallelic expression for CHD Exome (n=130) and CHD WGS (n=14) probands and GTEx donors (n=95). Standard deviations are indicated. Panel (b) shows that unfiltered allelically-biased SNPs have higher reference base expression at >80% of SNPs (hatched bars), indicating likely alignment biases. SNPs in cardiovascular imprinted genes (white bars) and extreme ASE SNPs (gray bars) are unbiased (i.e. reference base expression of allelically-biased SNPs approaches 50%). Genes that contain a single, "heterozygous" SNP with 100% monoallelic expression have a strong reference base expression bias (black bars), and are filtered out, due to a suspicious genotype. Panel (c) shows the false positive rate of calling ASE genes due to allele assignment of SNPs when phasing is not known. ASE genes were called on 1 out of every 18 F1 hybrid C57Bl6/Castaneus mouse SNPs (to more closely approximate human data, Supplementary Table 3) or on three human CHD subjects who were genotyped (along with their parents) by WGS. Fraction of false positive events were calculated as the difference in the number of ASE genes called in unphased data versus phased data, divided by the number of ASE genes called in phased data. False positive rates were generated for a minimum allele ratio of 4, 5, 6 and 7. The mouse (gray bars) and human (white bars) false positive rates are indicated; human false positive rate is 0 with a minimum allele ratio of 5 or greater.

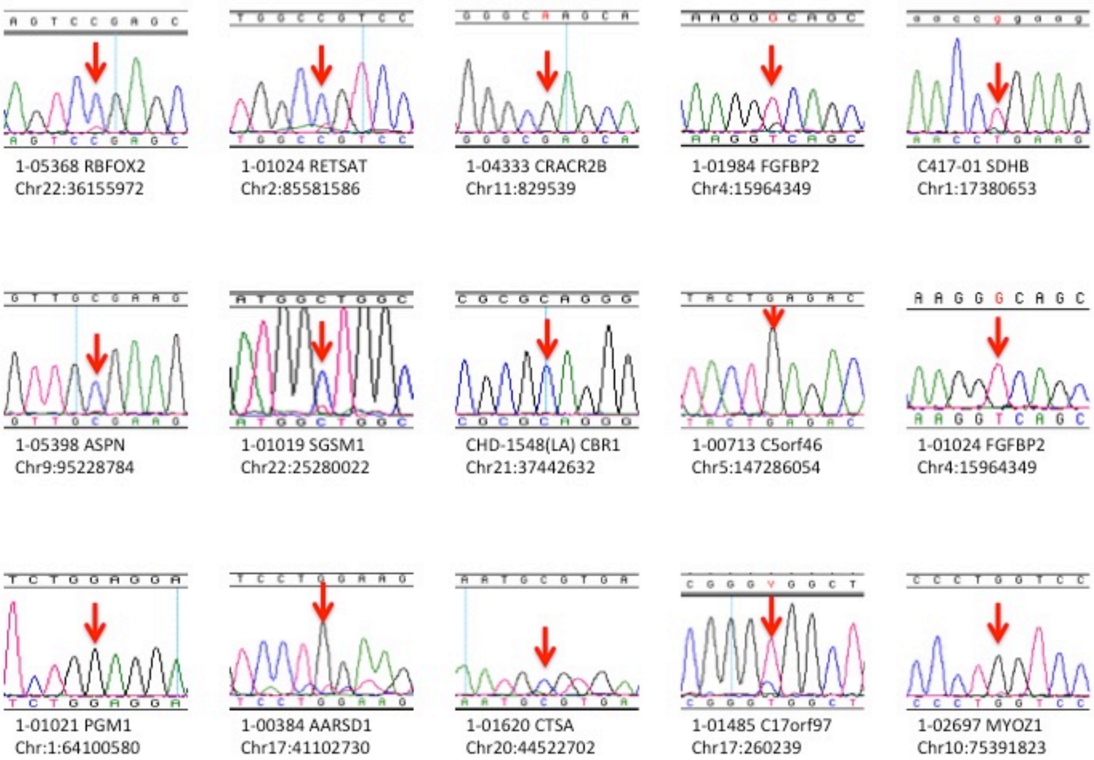

Supplementary Figure 3: ASE events confirmed by Sanger sequencing
Sanger sequencing traces of allelically-biased SNPs and eight surrounding nucleotides from extreme ASE events (shown are ASE events from Tables 2 and 3 only). For each trace, the subject, gene and chromosomal position are indicated.

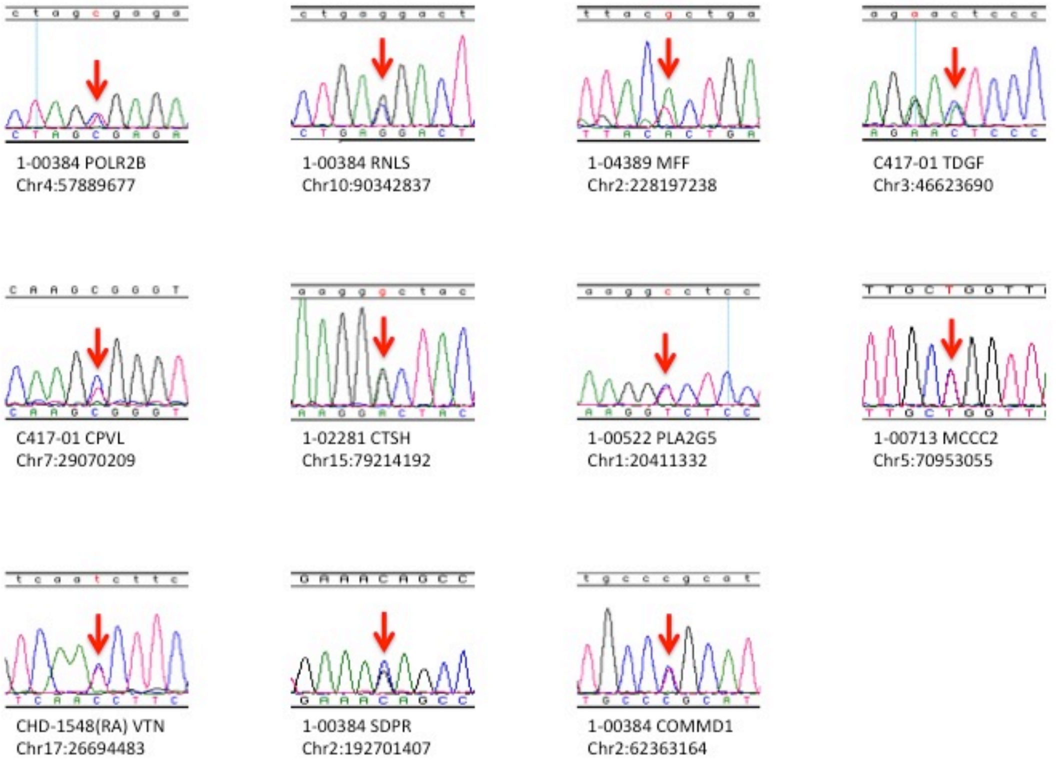

Supplementary Figure 4: False positive ASE events rejected by Sanger sequencing
Sanger sequencing traces of biallelically expressed SNPs and eight surrounding nucleotides from preliminarily called extreme ASE events. For each trace, the subject, gene and chromosomal position are indicated.

Supplementary Table 1: Number of different tissue samples, mean number of genes expressed per tissue per subject and mean number of expressed genes with at least one heterozygous SNP

Tissue	Tissue Abbrev.	No of samples	Genes Expressed/ subject[a]	Genes Het/ subject[b]
Aorta	AO	8	15820.78	4062.78
Aortic valve	AoValve	2	15429.50	3543.50
Atrial septum	AtrSpt	6	15537.33	3917.50
Ductus arteriosus	DuctArt	16	15620.76	3942.53
Interventricular septum	IVS	7	14938.00	3998.29
Left atrium	LA	5	15599.75	3551.25
Left ventricle	LV	13	15128.38	4046.38
Left ventricular outflow tract	LVOT	1	15997.00	2865.00
Papillary	Papillary	1	16406.00	3451.00
Pulmonary artery	PA	12	15883.36	3917.07
Pulmonary valve	PV	5	15967.71	4453.29
Right atrium	RA	50	15488.92	3958.30
Right ventricle	RV	42	15159.86	3817.43
Sub-aortic membrane	SubAoMembr	2	17651.00	3676.00
Unknown atrial	UnkAtrial	1	15231.00	2616.00
Unknown cardiac muscle	UnkCardMusc	1	16376.00	3688.00

[a]Average number of genes with expression > 2rpm (reads per million aligned reads)

[b]Average number of expressed genes containing heterozygous SNPs

Supplementary Table 2: Number of heterozygous SNPs and extreme ASE events filtered per sample

	Heterozygous SNP filters				ASE event filters		
**Filters[a]**	**CHD WGS**	**CHD Exome**	**GTEX**		**CHD WGS**	**CHD Exome**	**GTEX**
Total expressed heterozygous SNPs or preliminary ASE events	9310.1	4833.0	6070.9		31.2	19.1	21.6
I. Exclusion of SNPs							
A) Low-quality genotype called SNPs	1435.3	465.9	3.2		3.1	5.0	3.6
B) SNPs in alternatively spliced exons	36.1	33.0	40.2		1.6	0.6	2.0
C) SNPs with expected alignment biases	397.1	92.6	100.0		4.1	1.3	0.9
D) SNPs biased in multiple subjects	27.1	34.1	45.2		3.4	5.2	7.1
II. Exclusion of genes/RNAs							
A) Genes typically associated with ASE	129.7	42.1	34.4		4.4	3.7	3.7
B) RNAs with misaligned reads	815.1	528.8	266.8		7.8	2.3	0.7
C) Genes with low fetal heart expression	1.7	0.1	1.2		0.1	0.0	0.3
D) Known imprinted genes	65.3	33.6	55.8		1.8	0.5	1.3
III. Quality control confirmation							
A) ASE not confirmed in fastq sequencing reads	7.0	3.8	6.8		2.3	0.2	0.8
B) Visual inspection of RNAseq data in IGV	1.6	0.9	1.7		0.6	0.1	0.6
C) Sanger sequencing	1.4	0.0	NA		0.6	0.0	NA
Total filtered SNPs or extreme ASE events	2917.4	1234.9	555.3		29.8	18.9	21.0
Total biallelic SNPs	6388.8	3596.5	5511.3		NA	NA	NA
Total passing SNPs or extreme ASE events	3.9	1.6	4.3		1.4	0.2	0.6

[a]Filters are described in detail in Methods

Supplementary Table 3: C57Bl6/Castaneus F1 mouse RNAseq Data

F1 hybrid	RNAseq Tissues	Unique Aligned Reads	Expressed Genes[a]	Expressed Het SNPs (1 of 18 SNPs)[b]	Expressed Genes with Het SNPs (1 of 18 SNPs)[c]
C57Bl6 x CAST	LA	35355562	14549	99362 (9250)	12463 (6468)
C57Bl6 x CAST	Liver	33983102	14776	104377 (9593)	12765 (6665)
C57Bl6 x CAST	LV	33981526	14196	100756 (9144)	12192 (6317)
C57Bl6 x CAST	PA	36119922	15136	107543 (9818)	13102 (6817)
C57Bl6 x CAST	RA	33468184	14667	94928 (9116)	12443 (6433)
C57Bl6 x CAST	RV	35060410	14336	97199 (9116)	12317 (6380)
C57Bl6 x CAST	SKM	30808494	15089	101565 (9669)	12956 (6756)

See Supplemental Table 1 for tissue abbreviations; SKM, Skeletal muscle

[a]Number of genes with expression > 2rpm (reads per million aligned reads)

[b]Number of heterozygous SNPs with a minimum of 1 read

[c]Number of genes with expression > 2rpm, containing a heterozygous SNP

Supplementary Table 4: ASE genes over-represented (>5% ASE; > 1 ASE subject) in CHD cases and/or GTEX controls

**Gene**	**CHD ASE**[a]	**GTEX ASE**[a]	**% ASE**	**FHE**[b]	**PCGC cases LOF AF**[c]	**PCGC controls LOF AF**[c]	**ExAC LOF AF**[c]
ACSM5	2/6	1/2	37.50	2.4	0	1.1E-03	1.9E-03
AKR1C1	1/11	1/1	16.67	14.8	1.9E-03	8.3E-04	8.1E-04
CECR1	1/94	7/58	5.26	62.0	0	0	8.2E-05
ERAP2	0/5	2/15	10.00	4.52	3.0E-03	1.9E-03	2.1E-03
GLP1R	1/35	3/29	6.25	43.3	0	0	1.1E-04
MRPS26	0/4	3/11	20.00	51.7	0	0	9.1E-05
MYH6	5/17[d]	0/0	29.41	3646	7.5E-04	5.6E-04	6.9E-04
MYL7	1/16	1/13	6.90	3228	3.7E-04	2.8E-04	1.5E-04
NR1H2	0/15	3/19	8.82	62.6	0	2.8E-04	5.5E-05
PPP1R3A	3/44	1/22	6.06	4.7	1.1E-03	2.8E-03	3.5E-03
PRSS50	1/9	3/15	16.67	17.9	3.7E-04	0	1.3E-03
S100A12	0/2	1/18	5.00	3.1	3.7E-04	0	1.8E-05
THNSL2	0/10	1/5	6.67	8.43	3.4E-03	1.4E-03	4.0E-03
TNNT3	0/29	3/23	5.77	153.7	3.7E-04	0	5.5E-05
UQCRFS1	0/12	4/16	14.29	95.8	3.7E-04	0	9.1E-06
VWDE	0/9	1/10	5.26	3.14	2.4E-02	1.6E-02	5.5E-03

FHE, Fetal heart expression; LOF AF, Loss-of-function allele frequency

[a]Number of subjects with ASE /number of informative subjects

[b]Gene specific reads per million aligned reads

[c]Allele frequency for rare (MAF < 0.01), loss-of-function variants that are not alternatively spliced

[d]ASE observed only in extra-cardiac tissues, so 5/17 refers to just extra-cardiac tissues

Supplementary Table 5: ASE genes with low fetal heart expression

Gene	CHD ASE[a]	GTEX ASE[a]	% ASE	FHE[b]	PCGC cases LOF AF[c]	PCGC controls LOF AF[c]	ExAC LOF AF[c]
MYH1	0/1	2/6	28.57	0.5	7.5E-03	4.2E-03	9.0E-03
C6	0/53	1/72	0.80	0.9	2.6E-03	5.6E-04	3.4E-03
PGPEP1L	1/4	0/15	5.26	0.6	3.4E-03	8.3E-04	3.0E-03
DMBT1	0/1	1/1	50.00	0.3	1.5E-03	1.1E-03	2.8E-03
LMAN1L	0/24	1/33	1.75	1	3.0E-03	1.7E-03	2.8E-03
CYP4F12	0/18	1/35	1.89	0.6	7.5E-04	2.2E-03	2.6E-03
KLKB1	0/23	1/31	1.85	0.2	1.5E-03	5.6E-04	2.2E-03
LCNL1	1/21	0/27	2.08	0.5	8.2E-03	5.0E-03	1.9E-03
DNAAF1	0/0	1/2	50.00	1.9	3.4E-03	1.7E-03	1.9E-03
OTOGL	0/20	1/18	2.63	0.7	1.5E-03	2.5E-03	1.8E-03
UGT2B4	1/16	0/1	5.88	0.6	3.7E-04	5.6E-04	1.4E-03
APOB	1/12	0/41	1.89	1.4	7.5E-04	1.1E-03	9.0E-04
CYP4F11	0/3	1/2	20.00	0.9	1.1E-03	2.8E-04	8.4E-04
ABCC8	0/26	1/46	1.39	1.6	3.7E-04	5.6E-04	7.3E-04
CYP4B1	0/8	1/39	2.13	0.3	1.1E-03	5.6E-04	5.9E-04
SPINK5	1/12	0/18	3.33	0.7	0	5.6E-04	5.3E-04
CST7	0/13	1/34	2.13	0.8	0	5.6E-04	4.5E-04
GRIN2C	0/21	1/22	2.33	1.3	7.5E-04	0	3.1E-04
C1orf116	0/1	1/2	33.33	0.4	0	5.6E-04	2.4E-04
PNLIP	0/0	1/1	100.00	0	1.1E-03	2.8E-04	1.6E-04
C4BPA	0/0	1/3	33.33	0.2	0	0	1.3E-04
CA3	0/20	1/41	1.64	1.2	0	0	1.3E-04
CLEC12A	0/5	3/17	13.64	0.4	0	5.6E-04	1.2E-04
SSTR5	0/2	1/28	3.33	0	0	2.8E-04	1.2E-04
MLPH	2/74	0/50	1.61	1.5	3.7E-04	2.8E-04	1.1E-04
REG1A	0/0	1/1	100.00	0	0	2.8E-04	6.4E-05
GJD2	0/2	1/12	7.14	0	0	0	4.5E-05
LOR	0/0	1/18	5.56	0	0	0	2.7E-05
NPY	0/5	1/9	7.14	1.3	0	0	1.8E-05
INHBB	0/2	1/15	5.88	0.5	0	0	0

FHE, Fetal heart expression; LOF AF, Loss-of-function allele frequency

[a]Number of subjects with ASE /number of informative subjects

[b]Gene specific reads per million aligned reads

[c]Allele frequency for rare (MAF < 0.01), loss-of-function variants that are not alternatively spliced

Supplementary Table 6: Genes with ASE in CHD Subjects (excluding imprinted genes)

Blind ID	ASE Tissue(s)	Gene	Chr	Allele 1 reads[a]	Allele 2 reads[a]	Num SNPs	Allele Bias	Pval[b]	CHD ASE Subjects[c]	GTEX ASE Subjects[c]	% ASE	FHE[d]	Tissue Specific	LOF mutation (silenced allele)[e]	LOF mutation (expressed allele)[f]	*de novo*[g]	Altered expression[h]	PCGC cases LOF AF[i]	PCGC controls LOF AF[i]	ExAC LOF AF[i]
1-00384	IVS	AARSD1	17	33	4	1	8.3	2.3E-03	1/29	0/17	2	138		1			Loss	0	0	0
1-00618	RA	ACOX2	3	36	0	2	∞	6.1E-08	2/60	0/21	2	28.5						1.9E-03	2.2E-03	2.8E-03
1-04724	LV	ACOX2	3	35	0	3	∞	1.1E-07	2/60	0/21	2	28.5						1.9E-03	2.2E-03	2.8E-03
1-04119	PA	ARSA	22	31	3	2	10.3	1.4E-03	1/112	0/61	1	75.9						3.7E-04	8.3E-04	1.0E-03
1-05398	DuctArt	ASPN	9	44	1	1	44.0	5.1E-09	1/10	0/13	4	16.9		1				3.7E-04	1.1E-03	3.5E-04
1-00713	IVS,LV,RV	C17orf97	17	108	4	2	27.0	5.2E-24	2/12	0/8	10	16.8						7.5E-04	8.3E-04	4.1E-03
1-01485	RA	C17orf97	17	30	1	3	30.0	6.1E-05	2/12	0/8	10	16.8			1			7.5E-04	8.3E-04	4.1E-03
1-00713	LV,RV	C5orf46	5	61	5	1	12.2	5.3E-10	1/23	0/38	2	15.5					Loss	0	0	9.1E-05
1-00070	IVS,LV,RA	C7	5	49	3	1	16.3	1.9E-08	3/119	0/76	2	157		1				2.6E-03	5.6E-04	1.4E-03
1-01484	AtrSpt	C7	5	32	2	1	16.0	1.6E-04	3/119	0/76	2	157						2.6E-03	5.6E-04	1.4E-03
1-01984	LA,LV,RV	C7	5	76	8	1	9.5	8.7E-12	3/119	0/76	2	157	1 (not in RA)					2.6E-03	5.6E-04	1.4E-03
1-04173	PA	CASQ2	1	27	0	3	∞	2.7E-05	1/102	0/75	1	398						3.7E-04	2.8E-04	2.5E-04
CHD-1548	LA,LV,RA,RV	CBR1	21	38	1	1	38.0	4.0E-07	1/52	0/36	1	26.3					Loss	0	2.8E-04	2.6E-03
1-04050	RV	CDH13	16	43	4	1	10.8	6.1E-06	1/63	0/58	1	29.8						0	0	9.1E-06
1-01021	RA	CKMT2	5	426	47	1	9.1	3.2E-74	1/8	0/0	13	307						0	5.6E-04	4.8E-04
1-04333	RA	CRACR2B	11	21	0	2	∞	2.2E-03	1/59	0/55	1	20.9		1				3.7E-04	8.3E-04	5.4E-04
1-01620	PV	CTSA	20	73	3	1	24.3	4.9E-15	1/31	0/15	2	122		1				3.7E-04	2.8E-04	6.9E-04
1-00738	RV	DNAJC15	13	26	2	2	13.0	4.3E-03	1/46	0/74	1	112						7.5E-04	5.6E-04	2.5E-04
1-04136	DuctArt	FAM118A	22	175	6	1	29.2	5.9E-41	1/73	1/37	2	34.1						7.5E-04	5.6E-04	6.4E-04
1-01024	RA	FGFBP2	4	27	2	2	13.5	3.5E-03	2/16	1/32	6	0					Gain	1.1E-03	5.6E-04	4.5E-04
1-01984	IVS,LA,LV,RA,RV	FGFBP2	4	31	3	2	10.3	1.4E-03	2/16	1/32	6	0			1		Gain	1.1E-03	5.6E-04	4.5E-04
1-00713	IVS,LV,RV	FRA10AC1	10	35	1	2	35.0	2.3E-06	1/98	0/64	1	64.1						0	5.6E-04	3.2E-04
1-02073	AO	GBP3	1	26	2	23	13.0	5.7E-03	1/60	0/53	1	3.0						1.9E-03	1.9E-03	8.9E-03
1-05517	UnkCardMsc	GGA3	17	33	4	1	8.3	1.8E-03	1/28	0/20	2	51.3						0	0	1.0E-04
1-03051	AO	GTF2IRD2	7	22	0	2	∞	1.2E-03	1/26	0/0	4	10.9						7.5E-04	0	3.5E-04
1-01019	RA	KIF1A	2	228	14	6	16.3	1.4E-47	1/68	0/17	1	218						0	2.8E-04	7.3E-05
1-00425	DuctArt	MB	22	21	0	2	∞	1.7E-03	1/67	0/50	1	1044						0	2.8E-04	0
1-01446	RA	MYOZ1	10	294	16	1	18.4	4.5E-64	2/10	0/3	15	4.5	1 (not in PA)					0	2.8E-04	3.5E-04
1-02697	RV	MYOZ1	10	27	0	1	∞	3.6E-05	2/10	0/3	15	4.5	1 (not in PV)				Gain	0	2.8E-04	3.5E-04
1-01620	PV	PBXIP1	1	46	1	1	46.0	1.8E-09	1/73	1/64	1	113.7						0	2.8E-04	5.7E-04
1-01021	RA	PGM1	1	44	3	2	14.7	4.8E-07	1/114	0/73	1	124		1				3.7E-04	0	6.4E-05
1-02845	RA	PRSS35	6	85	8	1	10.6	4.4E-14	1/30	0/5	3	140						3.7E-04	2.8E-04	4.2E-04
1-05846	RV	RASGRP3	2	62	4	2	15.5	3.1E-11	1/67	0/61	1	37.6						0	0	2.9E-04
1-05368	DuctArt	RBFOX2	22	35	1	1	35.0	2.1E-06	1/1	0/0	100	235		1		1	Loss	1.1E-03	0	1.8E-05
1-01024	RA	RETSAT	2	62	2	1	31.0	5.1E-13	1/22	0/4	4	15.8		1		1		7.1E-03	5.6E-03	9.7E-03
C417-01	IVS,LV,RA	SDHB	1	24	1	1	24.0	3.8E-03	1/16	0/9	4	135					Loss	0	0	8.2E-05
C417-01	IVS,LV,RV	SERHL2	22	37	3	1	12.3	4.5E-05	1/26	0/0	4	11.1				1		9.7E-03	8.6E-03	1.6E-02
1-01019	RA	SGSM1	22	105	5	5	21.0	5.0E-22	1/50	0/14	2	9.9				1	Loss	3.7E-04	0	9.1E-05
1-04606	RA	SLC45A2	5	29	0	2	∞	7.3E-06	1/15	0/0	7	7.6						1.9E-03	2.8E-04	4.5E-04
1-04056	RV	TMEM176A	7	80	4	3	20.0	3.5E-16	1/113	1/70	1	35.2						0	2.8E-04	2.7E-04
1-00522	LV,RV	TMEM176B	7	78	4	2	19.5	1.0E-15	3/107	2/68	3	59.9						3.7E-04	0	2.0E-04
1-01484	AtrSpt	TMEM176B	7	98	9	1	10.9	1.1E-16	3/107	2/68	3	59.9						3.7E-04	0	2.0E-04
1-04056	RV	TMEM176B	7	50	5	2	10.0	3.3E-07	3/107	2/68	3	59.9	1 (not in DuctArt)					3.7E-04	0	2.0E-04
1-02697	RV	VTN	17	37	4	1	9.3	2.2E-04	2/53	0/54	2	6.9	1 (not in PV)					1.9E-03	1.7E-03	3.5E-03
C417-01	IVS,LV,RV	XRCC6BP1	12	47	5	1	9.4	2.9E-06	1/64	0/36	1	6.5						3.7E-04	2.8E-04	1.9E-04

FHE, Fetal heart expression; LOF AF, Loss-of-function allele frequency

[a]Summed reads of all ASE Tissue(s)

[b]Pval: Binomial distribution of Allele 1 and Allele 2 reads, corrected by the number of expressed genes containing heterozygous SNPs (Supplementary Data 2)

[c]Number of subjects with ASE /number of informative subjects

[d]Gene specific reads per million aligned reads

[e]Subject contains loss of function mutation (Nonsense, Splice Site, Frameshift) in silenced allele

[f]Subject contains loss of function mutation in expressed allele

[g]*de novo* loss of function mutation identified in this gene in a different CHD subject

[h]Proband expression compared to mean expression for each tissue; Loss of allele expression ($p < 0.05$, fold < 0.65); Gain of expression ($p < 0.05$, fold > 5)

[i]Allele frequency for rare (MAF < 0.01), loss-of-function variants that are not alternatively spliced

Supplementary Table 7: Genes with ASE in GTEx Subjects

GTEx ID	ASE Tissue(s)	Gene	Chr	Allele 1 reads[a]	Allele 2 reads[a]	Num SNPs	Allele Bias	Pval[b]	CHD ASE Subjects[c]	GTEX ASE Subjects[c]	% ASE	FHE[d]	Tissue Specific	*de novo*[e]	Altered expression[f]	PCGC cases LOF AF[g]	PCGC controls LOF AF[g]	ExAC LOF AF[g]
GTEX-UJMC	LV	ACTA1	1	962	3	1	320.7	2.7E-279	0/4	1/37	2	269.8				3.73E-04	0	1.36E-04
GTEX-WYJK	LV	ANPEP	15	40	4	2	10.0	3.6E-05	0/47	1/59	1	13.2	1			9.69E-03	8.06E-03	7.44E-03
GTEX-P4QS	LV	APOL4	22	72	0	8	∞	1.2E-18	0/32	1/15	2	29.8				0	5.56E-04	3.09E-04
GTEX-OOBJ	LV	ASPG	14	20	0	3	∞	5.0E-03	0/47	2/36	2	54.4				2.24E-03	8.33E-04	8.18E-04
GTEX-P4PQ	LV	ASPG	14	27	2	1	13.5	3.4E-03	0/47	2/36	2	54.4				2.24E-03	8.33E-04	8.18E-04
GTEX-WHWD	LV	ATP6V1C2	2	41	5	2	8.2	8.8E-05	0/3	1/31	3	28.1				3.73E-04	0	2.82E-04
GTEX-WH7G	RA	C11orf31	11	182	1	1	182.0	6.8E-50	0/7	1/1	13	133				7.46E-04	1.67E-03	3.12E-03
GTEX-W5X1	RA	C21orf56	21	34	0	2	∞	3.2E-07	0/22	1/14	3	12.3				7.5E-04	0	ND
GTEX-O5YT	LV	CLYBL	13	39	1	2	39.0	1.6E-07	0/50	1/29	1	32				1.49E-03	1.11E-03	9.36E-04
GTEX-U8XE	LV,RA	CRAT	9	81	10	1	8.1	1.2E-11	0/58	1/6	2	67				0	2.78E-04	3.00E-04
GTEX-QDVJ	LV	CRYZ	1	65	8	1	8.1	5.1E-09	0/101	1/67	1	25.3				3.73E-04	0	6.45E-04
GTEX-SUCS	LV	CTU2	16	41	3	2	13.7	3.2E-06	0/46	1/67	1	17.5				3.73E-04	8.33E-04	1.08E-03
GTEX-UJMC	LV	DDX19A	16	37	4	1	9.3	2.6E-04	0/65	1/27	1	53.7				3.73E-04	0	6.36E-05
GTEX-WZTO	LV	DUSP27	1	65	1	1	65.0	4.1E-15	0/91	1/66	1	144.6	1			2.61E-03	8.33E-04	1.70E-03
GTEX-WHPG	LV,RA	EIF2S2	20	526	1	1	526.0	6.0E-153	0/6	1/17	4	129				0	2.8E-04	2.7E-05
GTEX-UJMC	LV	ERICH1	8	25	1	1	25.0	2.2E-03	0/82	1/59	1	25.1				1.12E-03	2.78E-04	3.55E-04
GTEX-WYJK	LV,RA	ETFB	19	1646	2	1	823.0	0.0E+00	0/75	1/52	1	119				0	0	8.18E-05
GTEX-SIU7	LV	FAM118A	22	70	7	3	10.0	6.1E-11	1/73	1/37	2	34.1				7.46E-04	5.56E-04	6.36E-04
GTEX-NFK9	LV	FGFBP2	4	46	6	1	7.7	2.6E-05	2/16	1/32	6	0				1.12E-03	5.56E-04	4.55E-04
GTEX-OXRK	LV	GNPTG	16	131	1	1	131.0	1.1E-34	0/3	1/3	17	49.9				0	2.78E-04	4.45E-04
GTEX-WWYW	LV	GPIHBP1	8	29	3	2	9.7	5.2E-03	0/63	1/65	1	20.6	1			0	0	5.45E-05
GTEX-Q2AH	LV	IPO9	1	38	1	1	38.0	3.9E-07	0/20	1/24	2	204.3				0	0	6.36E-05
GTEX-WI4N	RA	KDELR3	22	33	3	1	11.0	5.0E-04	0/51	1/32	1	14.5				7.46E-04	2.78E-04	1.06E-03
GTEX-OHPM	LV	KRT10	17	46	5	1	9.2	4.5E-06	0/1	1/15	6	24.5				0	0	1.00E-04
GTEX-NFK9	LV	LGALS3BP	17	410	1	1	410.0	4.4E-118	0/34	1/23	2	216				7.46E-04	0	8.36E-04
GTEX-X3Y1	LV,RA	LRRC42	1	86	1	1	86.0	3.3E-21	0/39	1/26	2	39.3				1.86E-03	8.33E-04	4.55E-04
GTEX-OXRK	LV	MAP7D1	1	335	1	1	335.0	1.1E-95	0/72	1/16	1	154.8				3.73E-04	2.78E-04	6.55E-04
GTEX-WL46	RA	MYH7	14	186	24	1	7.8	6.3E-29	0/74	1/74	1	6221				0	0	2.73E-04
GTEX-WHSE	LV	NGRN	15	412	4	1	103.0	3.1E-113	0/23	2/23	4	165				3.73E-04	0	1.73E-04
GTEX-WK11	RA	NGRN	15	529	3	1	176.3	8.6E-150	0/23	2/23	4	165				3.73E-04	0	1.73E-04
GTEX-NFK9	LV	NPEPL1	20	97	2	1	48.5	4.3E-23	0/38	1/61	1	80.9				0	2.78E-04	1.45E-04
GTEX-UJMC	LV	NPPB	1	56	2	1	28.0	3.2E-11	0/6	1/2	13	122.7				0	2.78E-04	8.18E-05
GTEX-QEG5	LV	NQO1	16	48	6	1	8.0	6.2E-06	0/51	1/41	1	11.2				7.46E-04	5.56E-04	2.36E-04
GTEX-UJHI	LV	OBSL1	2	431	1	1	431.0	2.2E-124	0/138	1/83	0	178.3				0	5.56E-04	5.36E-04
GTEX-U4B1	LV	PBXIP1	1	101	5	2	20.2	5.6E-21	1/73	1/64	1	113.7				0	2.78E-04	5.73E-04
GTEX-NFK9	LV	PDXDC1	16	33	2	1	16.5	9.8E-05	0/113	1/52	1	116				2.98E-03	1.94E-03	5.23E-03
GTEX-WFON	LV,RA	PLOD1	1	50	4	1	12.5	7.5E-08	0/111	1/58	1	169.5				0	2.78E-04	3.00E-04
GTEX-UPK5	LV,RA	PPM1G	2	97	5	1	19.4	7.5E-20	0/1	1/3	25	58.7				0	2.78E-04	6.36E-05
GTEX-OIZH	LV	PPP2R2D	10	80	1	1	80.0	1.4E-19	0/66	1/35	1	27.2				0	0	1.82E-05
GTEX-WFON	LV,RA	PPP6R2	22	76	1	1	76.0	2.2E-18	0/118	1/40	1	47.6				0	2.78E-04	1.36E-04
GTEX-OHPK	LV	PRSS45	3	104	0	1	∞	2.8E-28	0/1	1/2	33	38.8				1.27E-02	1.00E-02	8.43E-03
GTEX-T2IS	LV	PSMD2	3	34	1	1	34.0	4.3E-06	0/51	2/33	2	151.1				0	0	8.18E-05
GTEX-WQUQ	LV,RA	PSMD2	3	418	2	1	209.0	1.5E-118	0/51	2/33	2	151.1				0	0	8.18E-05
GTEX-OIZF	LV	QRSL1	6	71	1	1	71.0	6.3E-17	0/91	1/53	1	26.1				3.73E-04	2.78E-04	2.82E-04
GTEX-WEY5	LV,RA	RAB3GAP1	2	103	9	2	11.4	4.6E-18	0/101	1/51	1	48				7.46E-04	2.78E-04	3.09E-04
GTEX-P4QS	LV	SCAMP5	15	51	3	2	17.0	8.0E-09	0/7	1/14	5	8.7				0	0	7.27E-05
GTEX-UJHI	LV	SCCPDH	1	65	5	2	13.0	5.8E-11	0/35	1/67	1	55.9				3.73E-04	2.78E-04	3.64E-04
GTEX-WFON	LV	SCN5A	3	47	5	1	9.4	2.5E-06	0/90	1/51	1	173.5				0	5.56E-04	2.55E-04
GTEX-NFK9	LV	SEPN1	1	77	2	1	38.5	2.9E-17	0/68	1/47	1	255.7				0	0	3.82E-04
GTEX-NFK9	LV	SLC16A3	17	26	0	2	∞	8.5E-05	0/5	1/4	11	130				1.12E-03	2.22E-03	5.75E-03
GTEX-S32W	LV	SPATA20	17	88	11	1	8.0	9.2E-13	0/122	1/71	1	79.6				1.12E-03	1.67E-03	1.29E-03
GTEX-OXRK	LV	SPPL2B	19	101	0	2	∞	1.8E-27	0/97	1/53	1	316				3.73E-04	0	4.55E-05
GTEX-WQUQ	LV,RA	STX18	4	81	1	1	81.0	8.1E-20	0/14	1/11	4	48.3				0	2.78E-04	1.55E-04
GTEX-RU72	LV	TMC8	17	24	1	2	24.0	3.0E-03	0/26	1/33	2	5.9				0	2.78E-04	9.45E-04
GTEX-S3XE	LV	TMEM176A	7	53	7	1	7.6	1.4E-06	1/113	1/70	1	35.2				0	2.78E-04	2.73E-04
GTEX-QDVN	LV	TMEM176B	7	183	22	1	8.3	1.7E-29	3/107	2/68	3	59.9				3.73E-04	0	2.00E-04
GTEX-S3XE	LV	TMEM176B	7	265	15	2	17.7	5.7E-57	3/107	2/68	3	59.9				3.73E-04	0	2.00E-04
GTEX-OXRL	LV	TOP2B	3	59	4	1	14.8	2.8E-10	0/78	1/38	1	162.2				0	0	1.27E-04
GTEX-OXRK	LV	TRIP10	19	236	6	1	39.3	1.7E-58	0/13	1/8	5	96.2				3.73E-04	2.78E-04	3.09E-04
GTEX-U3ZH	LV	UPK3B	7	29	3	1	9.7	5.3E-03	0/6	1/1	14	35.4				1.86E-03	1.67E-03	8.27E-04

FHE, Fetal heart expression; LOF AF, Loss-of-function allele frequency

[a]Summed reads of all ASE Tissue(s)

[b]Pval: Binomial distribution of Allele 1 and Allele 2 reads, corrected by the number of expressed genes containing heterozygous SNPs (Supplementary Data 4)

[c]Number of subjects with ASE /number of informative subjects

[d]Gene specific reads per million aligned reads

[e]*de novo* loss of function mutation identified in this gene in a different CHD subject

[f]Proband expression compared to mean expression for each tissue; Loss of allele expression ($p < 0.05$, fold < 0.65); Gain of expression ($p < 0.05$, fold > 5)

[g]Allele frequency for rare (MAF < 0.01), loss-of-function variants that are not alternatively spliced

Supplementary Table 8: Imprinted Genes that are Not ASE in Cardiovascular Tissues

Gene	Chr:Pos	CHD ASE[a]	GTEx ASE[a]	% ASE	RNA type	Expr Allele	FHE[b]	Mouse
TP73	chr1:3569128-3652765	0/3	0/0	0	C	M	3.6	N/S
DIRAS3	chr1:68511644-68516481	6/16	3/12	32	C	P	6.1	N/O
LRRTM1	chr2:80515480-80531487	0/14	0/0	0	C	P	3.9	N/S
GPR1	chr2:207040041-207082771	0/9	0/0	0	C	P	4.3	N/E
PHACTR2	chr6:143929316-144152322	0/23	0/26	0	C	M	69.9	N/I
IGF2R	chr6:160390130-160527583	0/111	1/74	1	C	M	114.8	I
SLC22A3	chr6:160769424-160876014	0/39	0/41	0	C	M	4.1	N/I
MAGI2	chr7:77646373-79082890	0/14	0/17	0	C	M	19.1	N/I
PPP1R9A	chr7:94536948-94925727	0/56	0/52	0	C	M	24.1	N/I
TFPI2	chr7:93515744-93520065	2/14	0/2	13	C	M	8.6	N/I
SGCE	chr7:94214535-94285521	0/1	0/0	0	C	P	59.8	I
CPA4	chr7:129932973-129964020	2/5	1/5	30	C	M	3.0	N/I
ZFAT	chr8:135490030-135725292	1/88	2/66	2	C	P	13.5	N/I
GLIS3	chr9:3824127-4300035	0/5	0/3	0	C	P	2.1	N/I
INPP5F	chr10:121485558-121588662	12/54	1/57	12	C	P	17.2	N/I
KCNQ1	chr11:2466220-2870340	3/71	1/53	3	C	M	43.0	N/I
CDKN1C	chr11:2904447-2906995	0/1	0/0	0	C	M	100.7	I
SLC22A18AS	chr11:2909326-2925175	0/1	1/2	33	C	M	3.4	N/O
SLC22A18	chr11:2920950-2946476	3/101	2/62	3	C	M	22.3	N/I
OSBPL5	chr11:3108345-3187969	5/107	1/67	3	C	M	29.4	N/I
WT1	chr11:32409321-32457081	0/14	1/30	2	C	P	5.0	N/I
ANO1	chr11:69924407-70035652	0/94	0/66	0	C	M	13.0	N/I
ZC3H12C	chr11:109963925-110042566	0/12	1/32	2	C	P	8.2	N/I
NTM	chr11:131240370-132206716	0/26	0/11	0	C	M	21.6	N/I
RBP5	chr12:7276279-7281466	0/30	0/24	0	C	M	2.0	N/O
RB1	chr13:48877882-49056026	0/1	0/1	0	C	M	15.7	N/I
UBE3A	chr15:25582395-25684175	0/9	0/9	0	C	M	65.6	N/I
ATP10A	chr15:25923859-26110317	1/74	2/40	3	C	M	2.1	N/I
NAA60	chr16:3493667-3536963	3/70	3/49	5	C	M	45.8	N/S
ZNF331	chr19:54024176-54083523	20/53	15/63	30	C	P	24.2	N/O
L3MBTL1	chr20:42136319-42179593	1/2	0/2	25	C	P	33.6	N/E

C, Coding; FHE, Fetal heart expression; I, Imprinted; M, Maternal; N/E, Not expressed
N/I, Not imprinted; N/O, No mouse ortholog; N/S, No SNPs; P, Paternal
[a]Number of subjects with ASE /number of informative subjects
[b]Gene specific reads per million aligned reads

Supplementary Table 9: ASE analysis of all genes containing a rare, nonsense mutation

Gene	Blind ID	Chr	Pos	Effect	MAF	Ref Base Reads	LOF Base Reads	Allele1 Reads[a]	LOF Allele Reads[b]	Allele Bias	Allele Pval[c]	LOF SNP Pval[d]
ASPN	1-05398	9	95228784	STOP_GAINED	4.1E-05	44	1	44	1	44.0	1.28E-12	1.28E-12
RBFOX2	1-05368	22	36155972	STOP_GAINED	0	35	1	35	1	35.0	1.05E-09	1.05E-09
CTSA	1-01620	20	44522702	STOP_GAINED	0	73	3	73	3	24.0	9.3E-19	9.30E-19
PGM1	1-01021	1	64100580	STOP_GAINED	0	9	0	44	3	15.0	1.15E-10	0.001953
CPNE3	1-05799	8	87549853	STOP_GAINED	4.1E-05	13	1	24	2	12.0	4.84E-06	0.000854
BCO2	1-00984	11	112085566	STOP_GAINED	5.7E-04	17	2	17	2	8.5	0.000326	0.000326
ECH1	1-01399	19	39308123	STOP_GAINED	2.4E-04	17	2	17	2	8.5	0.000326	0.000326
NOC2L	1-04943	1	889185	STOP_GAINED	0	15	2	25	3	8.3	1.22E-05	0.001038
ITGA11	1-02664	15	68599977	STOP_GAINED	0	22	2	85	12	7.1	1.35E-14	4.94E-05
EVC	1-02231	4	5811287	STOP_GAINED	8.1E-06	14	1	34	5	6.8	1.05E-06	0.000458
FAM189A	1-02281	9	72003220	STOP_GAINED	1.2E-04	19	4	19	4	4.8	0.002111	0.002111
FMO2	1-05673	1	171173121	STOP_GAINED	1.6E-03	61	14	130	27	4.8	1.82E-17	2.97E-08
AS3MT	1-02281	10	104632959	STOP_GAINED	8.2E-06	13	2	23	5	4.6	0.000732	0.006409
ASB15	1-04682	7	123267310	STOP_GAINED	7.7E-03	24	6	36	9	4.0	2.52E-05	0.000553
BCAM	1-02945	19	45315576	STOP_GAINED	0	20	10	86	17	5.1	1.13E-12	0.027982
SHMT1	1-02664	17	18243393	STOP_GAINED	0	8	3	15	3	5.0	0.009338	0.241699
FASTKD1	1-05538	2	170387886	STOP_GAINED	6.9E-03	6	1	28	6	4.7	0.000235	0.164063
ATP11B	1-04389	3	182602552	STOP_GAINED	0	7	1	16	4	4.0	0.004621	0.062500
ZC3H13	1-01984	13	46538115	STOP_GAINED	8.1E-06	10	1	230	59	3.9	1.94E-25	0.005371
SLC16A5	1-02090	17	73096839	STOP_GAINED	0	13	3	15	4	3.8	0.007393	0.008545
DNAJC4	1-01282	11	64000046	STOP_GAINED	4.1E-05	27	8	27	8	3.4	0.000685	0.000685
PI4KA	1-01446	22	21068776	STOP_GAINED	0	16	6	155	46	3.4	1.87E-15	0.017789
SEMA6C	CHD-1548	1	151108125	STOP_GAINED	2.4E-05	121	31	383	126	3.0	3.74E-31	1.04E-13
NEDD9	1-03956	6	11185691	STOP_GAINED	0	1	4	7	21	3.0	0.013233	0.468750
BDP1	1-01094	5	70811913	STOP_GAINED	0	1	0	20	7	2.9	0.006616	0.500000
MYOM2	1-00713	8	2026918	STOP_GAINED	4.5E-04	228	47	3518	1275	2.8	1.1E-239	4.37E-30
ANXA5	1-03185	4	122593712	STOP_GAINED	5.5E-04	51	18	51	18	2.8	2.92E-05	2.92E-05
HMGCL	1-04737	1	24147035	STOP_GAINED	2.4E-05	3	3	17	6	2.8	0.012034	0.312500
CMYA5	1-03973	5	79028492	STOP_GAINED	2.5E-05	11	2	486	178	2.7	1.99E-34	0.009521
MCC	1-02626	5	112458498	STOP_GAINED	4.1E-05	13	5	13	5	2.6	0.032684	0.032684
LZTR1	1-05368	22	21343948	STOP_GAINED	9.8E-05	12	5	25	10	2.5	0.010686	0.094421
ANKRD50	1-02846	4	125590331	STOP_GAINED	0	2	0	19	8	2.4	0.016541	0.250000
RNF213	1-02903	17	78333985	STOP_GAINED	0	3	1	17	7	2.4	0.020629	0.250000
ANKZF1	1-05254	2	220100305	STOP_GAINED	1.6E-05	16	4	31	13	2.4	0.008853	0.013862
ZDHHC4	1-02883	7	6624829	STOP_GAINED	2.4E-05	13	5	24	11	2.2	0.012143	0.032684
MMS19	1-04606	10	99237155	STOP_GAINED	0	26	12	26	12	2.2	0.00985	0.009850
ASB15	1-00618	7	123267310	STOP_GAINED	7.7E-03	18	5	31	15	2.1	0.021817	0.012034
TRIM22	1-02921	11	5730745	STOP_GAINED	4.4E-04	14	9	29	14	2.1	0.008911	0.097417
FAM189A	1-00618	9	72003172	STOP_GAINED	0	43	21	45	22	2.0	0.005514	0.006685
CROCC	1-03956	1	17257012	STOP_GAINED	1.9E-04	5	1	26	13	2.0	0.044324	0.281250
MC1R	1-00801	16	89986122	STOP_GAINED	6.7E-04	6	4	13	7	1.9	0.073929	0.205078
ERV3-1	1-04056	7	64453145	STOP_GAINED	0	5	4	13	7	1.9	0.073929	0.246094
C1orf86	1-05538	1	2125193	STOP_GAINED	1.6E-05	8	14	8	14	1.8	0.228717	0.228717
DNHD1	1-00842	11	6592054	STOP_GAINED	4.6E-04	1	2	10	17	1.7	0.12571	0.750000
ZNF211	1-01557	19	58153465	STOP_GAINED	3.5E-03	9	16	33	57	1.7	0.00341	0.060885
JMJD7-PL	1-02073	15	42134121	STOP_GAINED	0	7	11	14	24	1.7	0.035178	0.121399
KDSR	1-04276	18	61002545	STOP_GAINED	0	12	7	12	7	1.7	0.096107	0.096107
ZNF434	1-05673	16	3433131	STOP_GAINED	0	12	7	12	7	1.7	0.192215	0.192215
TRIM45	1-00425	1	117663561	STOP_GAINED	1.2E-03	8	3	14	9	1.6	0.097417	0.080566
C5orf45	1-00596	5	179264615	STOP_GAINED	4.8E-04	10	11	15	24	1.6	0.045731	0.168188
PRMT7	1-05589	16	68363008	STOP_GAINED	0	2	0	13	8	1.6	0.097032	0.250000
DNAJC11	1-01382	1	6705927	STOP_GAINED	0	13	14	17	26	1.5	0.047882	0.149446
TRIM45	1-02248	1	117663561	STOP_GAINED	1.2E-03	1	7	19	28	1.5	0.049548	0.031250
UBA3	1-03897	3	69105371	STOP_GAINED	8.1E-06	23	15	23	15	1.5	0.056284	0.056284
RABEPK	1-04389	9	127969843	STOP_GAINED	1.2E-04	7	2	15	10	1.5	0.097417	0.140625
INCA1	CHD-1548	17	4893541	STOP_GAINED	2.1E-03	45	19	58	38	1.5	0.030544	0.001418
AEBP1	1-02922	7	44151907	STOP_GAINED	0	10	7	14	10	1.4	0.1169	0.148376

Supplementary Table 9, continued

DPY19L2	1-03805	12	64061882	STOP_GAINED	1.5E-03	2	5	34	49	1.4	0.022717	0.164063
PRTFDC1	1-03956	10	25138812	STOP_GAINED	3.1E-04	18	13	18	13	1.4	0.288132	0.288132
DDX49	1-04050	19	19039030	STOP_GAINED	5.1E-03	3	3	11	8	1.4	0.144161	0.312500
ATF7IP2	1-05538	16	10574766	STOP_GAINED	7.1E-04	3	4	11	15	1.4	0.345386	0.820313
IQGAP2	C417-01	5	75964611	STOP_GAINED	0	1	1	17	12	1.4	0.096664	0.500000
MAP3K6	1-05453	1	27683163	STOP_GAINED	3.3E-05	8	11	20	26	1.3	0.079698	0.144161
C11orf21	1-05846	11	2321829	STOP_GAINED	5.1E-03	3	4	11	14	1.3	0.132841	0.273438
ACAD10	1-02697	12	112167654	STOP_GAINED	2.0E-04	12	10	12	10	1.2	0.308345	0.308345
CLK2	1-04652	1	155238112	STOP_GAINED	0	14	12	14	12	1.2	0.143911	0.143911
ABTB1	1-05445	3	127396573	STOP_GAINED	3.9E-03	10	8	10	8	1.2	0.166924	0.166924
ABCC8	1-00618	11	17434263	STOP_GAINED	5.7E-05	10	9	10	9	1.1	0.528591	0.528591
C5orf45	1-00842	5	179280392	STOP_GAINED	2.6E-03	11	10	11	10	1.1	0.336376	0.336376
CMBL	1-02697	5	10280729	STOP_GAINED	9.3E-04	6	6	12	11	1.1	0.322361	0.451172
BCO2	1-02726	11	112085566	STOP_GAINED	5.7E-04	5	5	14	13	1.1	0.149446	0.246094
SLC27A3	1-02920	1	153750338	STOP_GAINED	2.5E-04	12	11	12	11	1.1	0.16118	0.161180
CISH	1-05254	3	50645538	STOP_GAINED	1.6E-05	10	7	25	22	1.1	0.316204	0.445129
SIGLEC1	1-05254	20	3687141	STOP_GAINED	9.9E-03	2	1	10	9	1.1	0.528591	1.125000
MLKL	1-05517	16	74708900	STOP_GAINED	1.6E-04	20	19	20	19	1.1	0.125371	0.125371
ZNF30	CHD-1548	19	35435364	STOP_GAINED	8.1E-06	10	11	10	11	1.1	0.504564	0.504564
FAM118A	1-02664	22	45728582	STOP_GAINED	0	148	152	148	152	1.0	0.134461	0.134461
MAST4	1-04333	5	66461716	STOP_GAINED	0	5	6	13	13	1.0	0.154981	0.225586

LOF, Loss-of-function; MAF, Minor allele frequency

[a]Summed reads for allele not containing LOF mutation

[b]Summed reads for allele containing LOF mutation

[c]Pval: Binomial distribution of Allele 1 and Allele 2 reads, corrected by the number of expressed genes (per sample) containing heterozygous nonsense mutations

[d]Pval: Binomial distribution of LOF SNP reads, corrected by the number of expressed genes (per sample) containing heterozygous nonsense mutations

Variants above black line are considered ASE genes (Allele bias > 4; Allele Pval and LOF SNP Pval both < 0.01)

Supplementary Table 10: Quantitative PCR confirmation of biallelic loss of expression

**Blind ID**	**Tissue**	**Gene**	**ΔΔCt**	**p-value**
1-03051	AO	LBH	2.63	4.40x10-11
1-03316	AO	ZBTB16	3.52	1.29x10-9
1-04119	PA	FRG1B	1.63	9.53x10-5
1-00596	RA	PHKG1	4.09	1.83x10-7
1-02921	RV	IRX5	1.71	0.005
1-02921	RV	TRMT2B	8.64	4.56x10-10

Supplementary Table 11: RTL1 maternally expressed in cardiovascular tissues

**Blind ID**	**Num SNPs**	**Maternal RTL1 allele reads[a]**	**Paternal RTL1 allele reads[a]**
1-00801	1	1	0
1-01119	1	1	0
1-02073	1	2	0
1-02812	1	1	0
1-02829	3	4	0
1-02969	2	2	0
1-03948	1	1	0
1-04056	1	1	0
1-04119	1	2	0
1-04173	1	1	0
1-04276	1	1	0
1-04389	1	1	0
1-04652	2	4	0
1-04947	1	1	0
1-05476	3	5	0
1-05517	1	5	0
1-05538	1	1	0
CHD-1548	1	10	0

[a]Summed reads of all proband tissues

Supplementary Table 12: Quantitative PCR primers for confirmation of biallelic loss of expression		
**Gene**	**Primer1**	**Primer2**
LBH	CAGTCAGTCCAGGTTGACCC	ATGAACCACTCTCTGCAGCC
ZBTB16	GATTTGCTAACCTCGCAGCAG	ACCATGATGACCACATCGCA
FRG1B	CCACACAAAGAAGAATCGCCC	TGTCCCCTGCTTCATTGCAT
PHKG1	GGGCACCCCAACATCATACA	GTCCACCTCTTTCCCGTAGC
IRX5	AGCATCTTGGCAGGACCTTT	GGCTGGTACAAGTAGCCCTG
TRMT2B	CCGCCTCAGAAAACTCCCTAC	AGGGAAGCAGTCCTGGTTTG
ACTB	CGCCGCCAGCTCACCATG	CACGATGGAGGGGAAGACGG
GAPDH_1	CCCTTCATTGACCTCAACTACATG	TGGGATTTCCATTGATGACAAGC
GAPDH_2	ACAGTCCATGCCATCACTGCC	GCCTGCTTCACCACCTTCTTG
GUSB	ATCGCCATCAACAACACA	CTTGGGATACTTGGAGGTG
PPIA	GAGGAAAACCGTGTACTATTAGC	GGGACCTTGTCTGCAAAC
